# Between‐year and spatial variation in body condition across the breeding cycle in a pelagic seabird, the Red‐billed Tropicbird

**DOI:** 10.1002/ece3.10743

**Published:** 2023-12-27

**Authors:** Annalea Beard, Robert J. Thomas, Renata Medeiros Mirra, Elizabeth Clingham, Leeann Henry, Sarah Saldanha, Jacob González‐Solís, Frank Hailer

**Affiliations:** ^1^ Organisms and Environment, School of Biosciences Sir Martin Evans Building, Cardiff University Cardiff Wales UK; ^2^ School of Dentistry Cardiff University, University Dental Hospital Cardiff Wales UK; ^3^ Environmental Management Division, Environmental, Natural Resources & Planning Portfolio St Helena Government St Helena Island, South Atlantic Ocean UK; ^4^ Ciències Ambientals, Facultat de Biologia Universitat de Barcelona (UB) Barcelona Spain; ^5^ Institut de Recerca de la Biodiversitat (IRBio) Universitat de Barcelona (UB) Barcelona Spain

**Keywords:** behaviour, body mass, chick growth, incubation shift, *Phaethon aethereus*, seabird ecology

## Abstract

Body condition in pelagic seabirds impacts key fitness‐related traits such as reproductive performance and breeding frequency. Regulation of body condition can be especially important for species with long incubation periods and long individual incubation shifts between foraging trips. Here, we show that body condition of adult Red‐billed Tropicbirds (*Phaethon aethereus*) at St Helena Island, South Atlantic Ocean, exhibited considerable variation between years (2013–2017) and between different stages of the breeding cycle. Females took the first incubation shift following egg laying, after which males and females alternated incubation shifts of varying length, ranging from <1 to 12 days. Body condition declined in both sexes during an incubation shift by an average of 22 g (2.83% of starting mass) per day and over the incubation period; mass loss was significantly greater during longer incubation shifts, later within a shift and later in the total incubation period. There was also significant differences in incubation behaviour and body condition between years; in 2015, coinciding with a moderate coastal warming event along the Angolan‐Namibian coastlines, adults on average undertook longer incubation shifts than in other years and had lower body condition. This suggests that substantial between‐year prey fluctuations in the Angola Benguela upwelling system may influence prey availability, in turn affecting incubation behaviour and regulation of body condition. Adults rearing chicks showed a significant reduction in body condition when chicks showed the fastest rate of growth. Chick growth rates during 2017 from two localities in the Atlantic Ocean: an oceanic (St Helena) versus neritic (Cabo Verde) population were similar, but chicks from St Helena were overall heavier and larger at fledging. Results from this multi‐year study highlight that flexibility and adaptability in body condition regulation will be important for populations of threatened species to optimise resources as global climate change increasingly influences prey availability.

## Graphical abstract

Body condition in Red‐billed Tropicbirds (*Phaethon aethereus*) at St Helena, South Atlantic Ocean varied considerably between years, affected by multiple different factors at different stages of the breeding cycle, likely influenced by the Angola Benguela upwelling system. Variation in chick growth rates at two regions in the Atlantic are also examined. Males on average had one day longer incubation shifts than females.

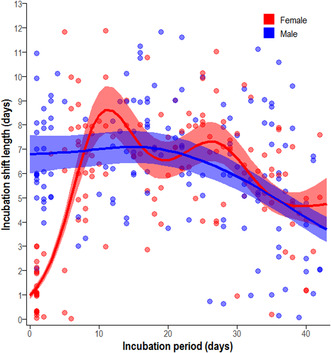

## 1 INTRODUCTION

Body condition in birds is a key factor underlying the success of breeding, migration and moult. In seabirds, parental body condition is known to influence and impact breeding and foraging regimes (Fisher, 1967; Labocha & Hayes, 2012; Quillfeldt et al., 2004). In pelagic seabirds, spending much of their time at sea and only returning to land to breed, it is challenging to understand the pressures acting upon them, such as the energetic costs of breeding and how they respond to these costs. While body condition and its regulation in Procellariiformes (albatrosses, petrels and shearwaters) and Sphenisciformes (penguins) have been examined in detail, for example (Chastel et al., 1995; Clay et al., 2023; Croxall, 1982; Croxall & Ricketts, 1983; Weimerskirch, 1990) Phaethontiformes (tropicbirds) have so far received little attention in this respect (Le Corre et al., 2003).

In seabirds, the division of breeding duties can influence breeding success. Males and females generally share breeding duties equally (Croxall & Ricketts, 1983), but differing parental investment between the sexes while breeding can occur in some species (Chaurand & Weimerskirch, 1994; Phillips, 1987; Pinet et al., 2012; Weimerskirch, 1995). Differences between sexes in breeding behaviour and regulation of body condition have been attributed to sexual size dimorphism (Lormée et al., 2003; Weimerskirch et al., 2006). Tropicbirds are typically monomorphic, with limited or no sexual size dimorphism (Nunes et al., 2013); however, sex differences in behaviour are also not uncommon in monomorphic seabirds (Lewis et al., 2002; Pinet et al., 2012).

Food resources are often scarce and patchily distributed in tropical marine environments (Abrams, 1985). While provisioning for their chick, pelagic seabirds must therefore find an equilibrium between reaching productive areas, searching for prey, returning to the chick at regular intervals to feed and/or guard while the partner forages and self‐provisioning (Sommerfeld & Hennicke, 2010). Parental provisioning frequency and meal size can influence chick growth rate, along with attendance at the nest providing temperature regulation and protection from predators and rival territorial conspecifics (Schreiber, 1994).

Bimodal foraging strategies (alternating long and short foraging trips) such as those employed by tropicbirds (Sommerfeld & Hennicke, 2010) are known to influence body condition (Weimerskirch, 1998; Welcker et al., 2012). This requires parent birds enduring extended fasting periods during incubation shifts, and balancing energy expenditure in provisioning for a chick with provisioning for themselves. Seabirds with long incubation periods typically lay a single egg clutch and fast while incubating while their partner is foraging at sea. The length of an incubation shift can substantially negatively affect adult body condition (Croxall, 1982), although the rate of loss of condition is influenced by individual differences in metabolic rates (Shoji et al., 2015). Chick provisioning periods in pelagic seabirds are also typically extended. These life history traits render species such as tropicbirds particularly vulnerable to unpredictable variations in food resources (Ballance & Pitman, 1999). There is a lack of knowledge of tropicbird chick growth rates, which have either been poorly documented and/or date from estimates obtained decades ago, when foraging conditions may have been very different (Fleet, 1974; Harris, 1969; Phillips, 1987; Stonehouse, 1962).

Chick growth and adult body condition can also vary among breeding seasons and different localities, due to inter‐annual variation in oceanographic conditions caused by El Niño Southern Oscillation (ESNO) and the Pacific Decadal Oscillation (PDO) which affect upwelling, ocean productivity and food availability, in turn leading to changes in parental foraging effort and nest attendance behaviour of seabirds (Blight et al., 2010; Castillo‐Guerrero et al., 2011; Champagnon et al., 2018; Polis et al., 1997; Schreiber, 1994). In the South Atlantic, south‐east trade winds create inter‐annual fluctuations of the Angola Benguela upwelling system, leading to warm ‘Benguela Niño’ and cold ‘Benguela Niña’ events (Feistel et al., 2003). Yet, in comparison with climate and oceanographic oscillations in the Pacific Ocean, where the effects have been extensively studied, little is known whether or how these oscillations might impact tropical south Atlantic ecosystems. Many seabird populations are located adjacent to continental shelves, where coastal upwelling systems can influence productivity, and thus local food availability can also influence adult body condition, breeding performance and foraging behaviour (Becker & Peery, 2007; Pereira et al., 2020; Simeone et al., 2002). In contrast, isolated oceanic populations that are out of reach of the coast or nearby land masses may be reliant on deep‐sea topographical features, such as seamounts for enhanced prey availability.

This study aimed to test several hypotheses about the role of body condition in mediating the breeding behaviour of Red‐billed Tropicbirds (*Phaethon aethereus*), the largest and least numerous tropicbird species with a globally declining population (McGehee, 2000). Firstly, we tested for between‐year differences in adult body condition, predicting that adults would exhibit poorer body condition in years of reduced food availability possibly mirroring variation in the environment among years. Secondly, we tested for changes in adult body condition between and within breeding stages, expecting that body condition would decline across the breeding season due to energetic constraints and that body condition would be negatively associated with the length of individual incubation shifts when the incubating bird is unable to forage. Thirdly, we tested the null hypothesis of equal parental incubation and provisioning effort between the two sexes, predicting that due to the limited sexual size dimorphism of Red‐billed Tropicbirds (Nunes et al., 2013) parental investment is likely to be similar between the sexes. Fourthly, we tested for differences in parental body condition at nests with differing reproductive outcomes (success or failure), expecting that breeding failure would be associated with poor parental body conditions. Finally, we compared chick growth rates between two localities in the Atlantic Ocean: an oceanic (St Helena) and neritic (Cabo Verde) population, predicting that because marine resources are more patchily distributed in large pelagic oceanic regions than in areas with predicable upwelling systems with higher prey availability, that chick growth rates would be lower in chicks from St Helena than in chicks from Cabo Verde.

## 2 METHODS

### 2.1 Study sites

**FIGURE 1:**
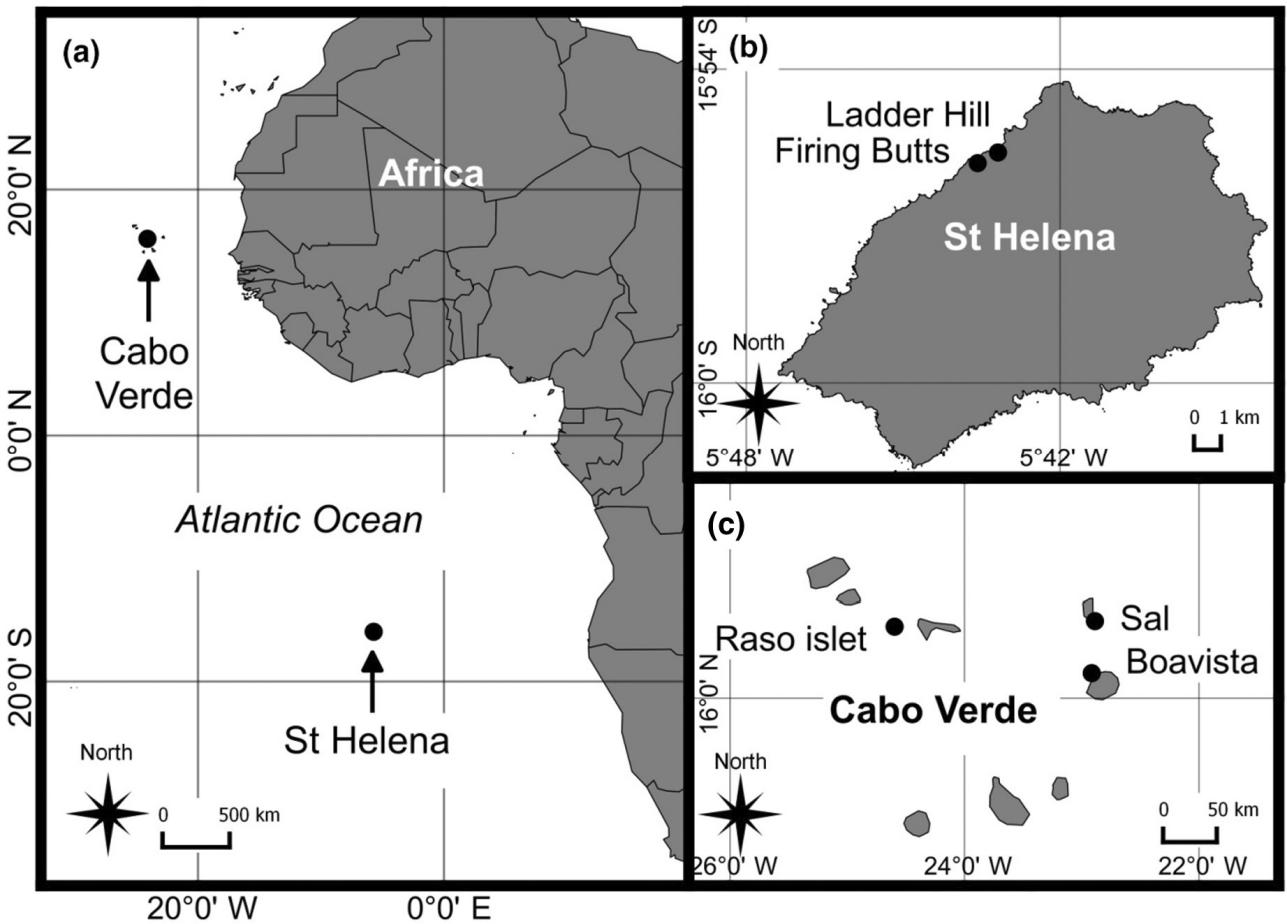
Location of the two study regions of Red‐billed Tropicbirds in (a) the tropical Atlantic Ocean, (b) St Helena and (c) Cabo Verde. Black circles indicate study sites.

This study was conducted in two regions of the Atlantic Ocean (Figure 1a): St Helena in the central south Atlantic (15°57′ S, 5°42′ W) and Cabo Verde in the central eastern Atlantic (16°00′ N, 24°00′ W). St Helena, a UK overseas territory, is an isolated subtropical oceanic island approximately 122 km^2^ in size, 1900 km west from the Angola/Namibia border (Figure 1a,b), located on the eastern edge of the South Atlantic subtropical Gyre in the path of the south‐east trade winds and the South Equatorial Current (Talley et al., 2011). Cabo Verde, consists of 10 volcanic islands that lie between 600 and 850 km west of Senegal, Africa (Figure 1a,c), located at the eastern boundary of the North Atlantic subtropical gyre at the southern limit of the Canary Current (Fernandes et al., 2005). The waters around St Helena are characterised by a generally low chlorophyll *a* concentration with substantial variation in spatial distribution between years (maximum range 0.3–0.5 mg/m^3^) (Thorpe et al., 2020), whereas Cabo Verde is considered a more productive oceanic region with more enriched waters (chlorophyll *a* concentration range 0.06–4.1 mg/m^3^ (Medrano et al., 2022; Silva, 2022; Vieira, 2019)).

### 2.2 Adult body condition

We obtained 1061 measures of adult body mass from 122 individuals as a reliable index of body condition (Labocha & Hayes, 2012). A minimum of 24 cavities per year were monitored between 21st August and 21st December, over a 5‐year period (2013–2017) at St Helena, using a combination of direct observations and infra‐red motion sensor cameras (see Appendix S1, Table S1 for details). Whenever possible, Bushnell Trophy Cameras were positioned near the entrance of cavities so that the arrival and departure of adults were continuously monitored. Cameras captured two pictures consecutively after the camera was triggered with a minimum interval of 5 s between successive triggers. All adults in cavities were caught by hand and fitted with a standard metal ring (issued by the British Trust for Ornithology; BTO) on their right tarsus, weighed and measured following Redfern and Clark (2001). To enable individual identification of breeding pairs without frequent capture or handling, any breeding adults were marked with a unique combination of colour marks on their head feathers (dyed with non‐toxic waterproof markers; Sharpie) under licence by the BTO (licence S5526). Each nest in a cavity was given a unique nest identification number to identify individual nesting attempts. Here we define pre‐breeding body condition as the mass of adults weighed within the 7‐day period prior to egg laying. Incubation body condition was defined as the mass of adults while sitting on an egg in a nest, chick rearing body condition as the mass of adults which were attending a chick on a nest and non‐breeding body condition as the mass of adults which were not known to be actively breeding at the time of weighing.

Fieldwork was conducted at two monitoring plots on St Helena (Ladder Hill and the Firing Butts) and two islands (Sal and Boavista) and one islet (Raso) in Cabo Verde, each containing accessible Red‐billed Tropicbird nesting cavities. Individual Red‐billed Tropicbirds usually have an annual breeding cycle, but the species can be found breeding throughout the year at both study sites, with a seasonal peak in productivity: October to May at Cabo Verde (Diop et al., 2019) and July to January at St Helena (Beard et al., 2023). Tropicbirds have an extended incubation period (~43 days; Stonehouse, 1962) with incubation shifts lasting up to 16 days (Fleet, 1974) and chick rearing period that can last 12–13 weeks before fledging (Boeken, 2016). Red‐billed Tropicbird populations were estimated to be up to 246 pairs at St Helena (Beard et al., 2023) but as few as 1100 pairs at Cabo Verde (BirdLife International, 2023).

Where possible, for each adult during incubation, we estimated the following covariates along with its weight: (1) The number of elapsed days of the incubation period (Incubation period days) when the adult was weighed. The incubation period (assuming up to 43 days (Harris, 1969)) was defined as the number of days from laying an egg to hatching a chick, based on known laying and/or hatch dates. (2) The total length (in days) of the individuals incubation shift (Incubation shift length). This was recorded from an incubating adults first day on the nest to the last day before the relieving mate returned to the nest. (3) The proportion of the individual incubation shift completed at the date of weighing (incubation shift proportion: day of incubation shift/Incubation shift length), as incubation shift length can vary during the incubation period. (4) Incubation outcome (hatching success: true/false). (5) The year the adult was monitored (2013–2017) and (6) the sex of the adult (male or female) which was determined following standard molecular sexing methods from feather samples collected during fieldwork (Fridolfsson & Ellegren, 1999). When weighing adults rearing chicks, we estimated the age of the chick (in days) based on the date the chick was first discovered hatched and/or known fledging dates, assuming a 93‐day chick rearing period (Harris, 1969) and the growth stage of plumage (Stonehouse, 1962).

### 2.3 Breeding regime

Incubation shifts at St Helena were calculated using a combination of (1) camera trap images with date and time stamp showing an adult returning to the cavity to change over incubation duties with its partner subsequently leaving the cavity, and when such images of a changeover event were not available (2) the mid‐point between two consecutive observations, where different adults were observed. Parental attendance during the chick rearing period was quantified by calculating the proportion of observations where at least one parent was present on the nest with the chick. A chick was deemed fledged if it was at least circa 85 days old (Boeken, 2016), known to weigh at least 500 g and absent from its cavity during subsequent observations, and/or camera images showed the chick leaving the cavity entrance at a near‐fledged stage.

### 2.4 Chick biometrics

Standard biometric measurements (weight: mass to the nearest gram using a Pesola spring balance, wing cord: flattened wing from the carpal joint to the tip of the longest primary, tarsus: maximum tarsus length from the distal point of the inter‐tarsal joint to the foot, head‐plus‐bill: bill trip to the posterior ridge formed by the parietal‐supraoccipital junction, culmen length: exposed culmen and nostril to bill: length from the distal end of the nostril to the trip of the bill using a calliper to the nearest 1 mm) were obtained following Redfern and Clark (2001) for all chicks at St Helena in the 2017 breeding season. Chick biometric data at Cabo Verde were pooled from two islands and one islet (Sal, Boavista and Raso respectively, Figure 1b) during the equivalent breeding season in 2017. A subset of biometrics (weight, wing, head and bill length, culmen length) was selected from Cabo Verde chicks with known hatch dates within a <15 day accuracy for a regional comparison of chick growth with St Helena. Estimated chick age since hatching and biometrics were recorded every second day at St Helena and a maximum of 5 days at Cabo Verde until either the breeding attempt failed, or the chick fledged.

### 2.5 Statistical analysis

All statistical analyses were carried out in the statistical program R 3.5.1 (R Development Core Team, 2018) implemented in R‐studio. All generalised linear mixed models (GLMMs) were implemented in the package *lme4* (Bates et al., 2015), all likelihood ratio tests (LRTs) in the *lmtest* package (Zeileis & Hothorn, 2002) and all generalised additive mixed models (GAMMs) in either *gamm4* package (Wood & Scheipl, 2020) or *mgcv* package (Wood, 2004), following Thomas and Lello (2017). Significant effects in final models were plotted using the R packages *ggplot2* (Wickham, 2016), *ggpubr* (Kassambara, 2020), *hrbrtheme* (Rudis, 2020). Estimated parameters for the growth curves were calculated in the R package *FlexParamCurve* (Oswald et al., 2012).

#### 2.5.1 Body condition

We tested for any effect of the monitoring period (Julian day) on adult body condition using a GAMM with a thin‐plate regression spline, gamma error family and log link function. To control for repeated measures of individuals and locations (pseudo‐replication), individual identity and cavity identity were both included as random effects.

We used GLMMs to test the hypothesis that body condition would vary by (1) sex, (2) breeding stage and (3) the interactive effect of sex and breeding stage. Body condition was treated as a normally distributed response variable using ‘log’ link function and also included individual identity and cavity identity as random effects in the model. For each variable (sex, breeding stage or sex:breeding stage), we constructed two models: One containing the variable as a fixed effect, and a null model without the variable as an effect, then compared the two models using a LRT (Lewis et al., 2011). We concluded that the variable was a significant predictor of body condition if the model containing the variable of interest was significantly better than the null model (threshold *p* < .05). We also tested for differences in body condition between years using the same model structure described above but included breeding stage as a fixed factor in both models.

We used GLMMs to assess whether body condition during the incubation and chick rearing breeding phase differed between sexes, years, breeding outcome (hatched: true/false, fledged: true/false) or date within the breeding phase. For body condition during the incubation period we included additional variables: the length of incubation shift: excluded incubation shifts that extended past the average incubation period (43 days: Harris (1969) in case incubation shift length was influenced by breeding outcome) and the proportion of incubation shift completed. For the chick rearing period, we also included the age of the chick (in weeks, up to 10 weeks of age) as a factor. For each variable and breeding stage, we constructed two models: One containing the variable as a fixed effect, and a null model. Given that the data comprised multiple body weights from the same individuals and nesting attempts, we included the random effects of individual identity (Ring ID) and nest identity (Nest ID) within each GLMM. A gamma distribution with a log link function was used to model the response variable (body mass). We compared the two models using a LRT as detailed above.

We then further explored which variables that were identified as significant from the LRTs (detailed above) best explained variation in body condition during incubation. To do this, we constructed a global model with each variable included as an additive term and as a two‐way interaction with each other variable. Model selection was performed by sequentially removing the least significant term until the model with the lowest Akaike's Information Criterion (AIC) value was identified (Burnham & Anderson, 2002).

#### 2.5.2 Incubation shifts

Descriptive statistics were generated for incubation shift frequency using a subset of data from pairs containing individuals of known sex, that were monitored from laying to hatching and where the shift sequence could be identified. We used GAMMs to explore whether variation in the time spent incubating an egg could be explained by any differences between sexes, years, hatching outcome or incubation periods. Given that our data were comprised of multiple incubation shifts from the same individuals and nesting attempts, we included the random effects of individual identity and nest identity. A gamma distribution with a log link function was used to model the response variable (Incubation shift length). Smoothed terms were fitted to the day of incubation period (Incubation period days) with a penalised regression spline (Thomas & Lello, 2017; Wood & Augustin, 2002). The full model included sex, hatching outcome and year as additive terms, as well as an interaction between sex and day of incubation period, hypothesising that incubation shift length may differ between successful and unsuccessful nests, years and sexes. The interactive effect of sex and day of incubation period was included, as tropicbirds are known to alternate incubation duties between sexes (Boeken, 2016). Model selection was based on a stepwise deletion process, by removing the least significant term at each step, evaluated by an ANOVA test and resulting change in AIC value until the model with the lowest AIC value was identified.

#### 2.5.3 Mass loss during incubation

The rate of mass loss is a reliable index of energy costs incurred by fasting birds during incubation (Croxall, 1982). The rate of mass loss during incubation was examined using the proportion of mass loss per day, calculated from a subset of masses during incubation from individuals that had more than one mass measurement during the same incubation shift. The difference between the two mass measures was divided by the number of days between measures, divided by the initial mass ×100, to give the proportional mass loss per day, for each individual. To investigate if the rate of mass loss during an incubation shift varied between years, sexes and between incubation outcome (hatching success: true/false) we then constructed two competing GLMMs with proportional mass loss per day as the response variable (one containing the variable as a fixed effect, and a null model without the variable as an effect, then comparing the two models using a LRT) to identify any effect of each variable (year, sex, incubation outcome) independently. We then explored if the variables identified as significant during incubation (proportion of incubations shift completed, incubation shift length, day of incubation period, defined above) also influenced proportional mass loss per day. For this, we constructed a global model from which the least significant terms were removed stepwise until the model with the lowest AIC value was identified.

#### 2.5.4 Parental attendance during chick rearing

Descriptive statistics on the proportion of visits where parents were present with chicks on the nest were generated to identify changes in parental attendance through the chick rearing period, firstly by the age of the chick in weeks and secondly by the age groups defined by Stonehouse (1962) to allow for a regional comparison with Ascension Island. Binomial tests were used to examine (1) any differences in overall attendance between St Helena and Ascension Island, and (2) differences in parental attendance between nests that failed and nests that successfully fledged a chick at St Helena, hypothesising that more attentive parents would have better breeding success.

#### 2.5.5 Chick growth

The growth rate and variation in chick body mass between the two regions was explored by fitting logistic growth curves (Richards, 1959) using the equation:

$$y=A/{\left(1+m\times e^{-k\times \left(\mathrm{age}-i\right)}\right)}^{l/m}$$

where *y* is the response variable (weight, wing, tarsus, head and bill, culmen and nostril to bill length), *A* represents the asymptote, *k* the rate at which the slope of the curve changes with age, *i* the inflexion point corresponding to the age at which the fastest growth is achieved, and *m* the shape parameter of the logistic curve (Richards, 1959).

To compare growth rates between geographical regions, we calculated the proportion of growth per day for each chick, firstly by calculating the difference in mass between two consecutive measures, then dividing the difference by the number of days between measures and by the initial mass. This gave the proportion of mass lost per day. A Gaussian GAMM with ‘identity’ link function was then used to test for effects of region on the proportion of growth by chick age. Nest identity was treated as a random effect to account for repeated measures from individual chicks and region as a fixed factor.

## 3 RESULTS

### 3.1 Influences on adult body condition

**FIGURE 2:**
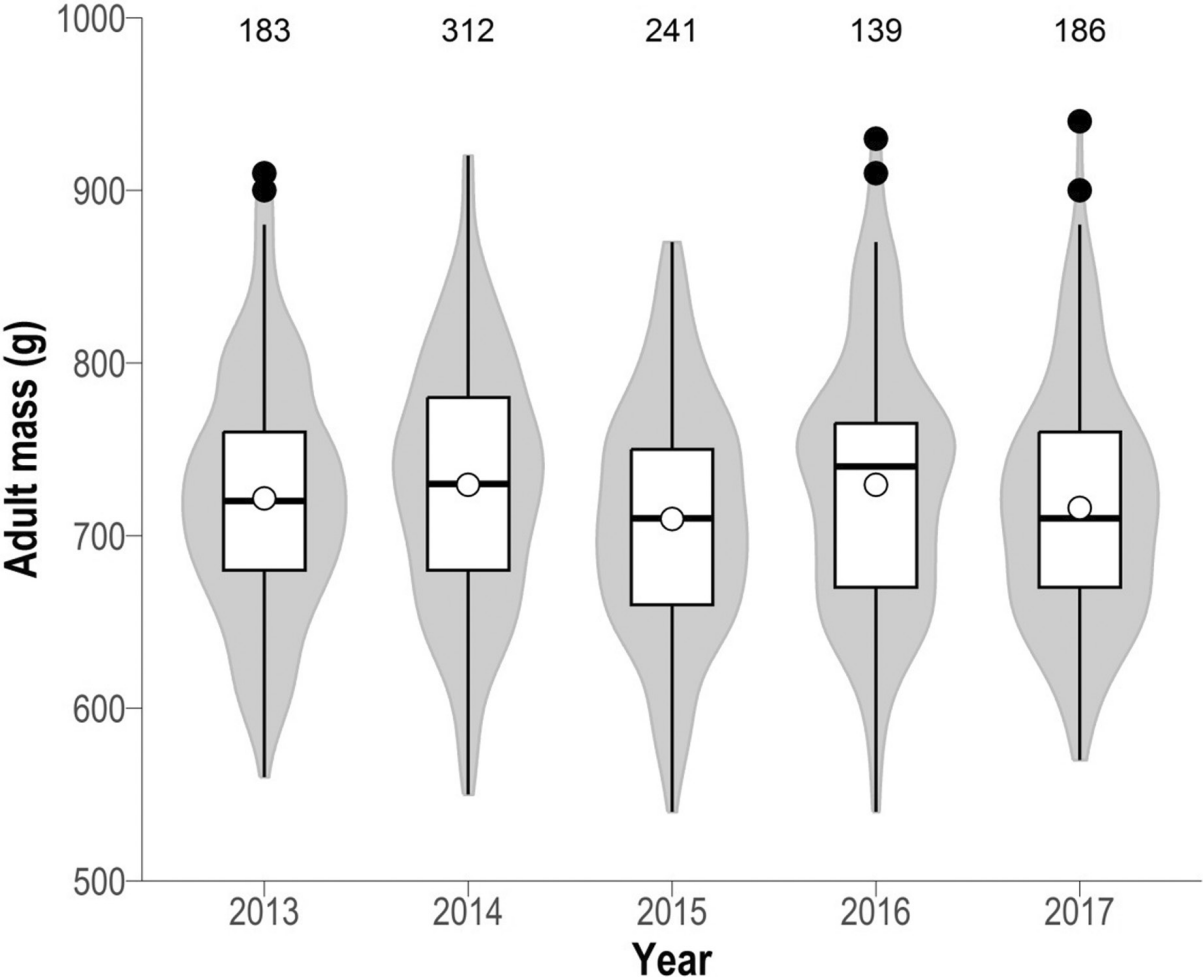
Temporal variation in body mass of adult Red‐billed Tropicbirds between 2013 and 2017 at St Helena Island, South Atlantic. Sample sizes are indicated at the top. Violin plots show the adult mass density distribution during each year. Black‐contoured boxplots show median (line), 25th and 75th percentiles (boxes), ranges within 1.5 times the height of the box (whiskers), outliers (black points) and means (white points). Sample sizes are shown at the top of the plot.

There was no significant linear association of adult body condition with Julian day (*p* = .251, *R* ^2^ = −.00169, *F* = 0.411, *n* = 1061), but pre‐breeding adults (*n* = 31) were on average 91 g heavier than adults rearing chicks and 70 g heavier than non‐breeding adults (LRT $\chi_{3}^{2}$ = 76.888, *p* < .001: Appendix S2, Figure S1). After accounting for differences between breeding stages, body condition also differed significantly between years (LRT $\chi_{4}^{2}$ = 11.261, *p* = .024): adults during 2015 (*n* = 241) were lighter (mean 710 ± 68 g) than in the other 4 years monitored (Figure 2). Although males were on average slightly heavier than females (728 ± 67 g, *n* = 554 and 714 ± 73 g, *n* = 507, respectively) there was no significant difference in body condition between sexes (LRT $\chi_{1}^{2}$ = 0.007, *p* = .933) or between the sexes during different breeding stages (interaction term: LRT $\chi_{3}^{2}$ = 3.2187, *p* = .359).

### 3.2 Changes in body condition during the incubation period

Body condition during incubation was significantly negatively associated with the length of the incubation shift being undertaken (incubation shift length: LRT $\chi_{1}^{2}$ = 4.481, *p* = .034), the proportion of the incubation shift they had completed (incubation shift proportion: LRT $\chi_{1}^{2}$ = 280.16, *p* < .001) and how far they were into their incubation period (incubation period days: LRT $\chi_{1}^{2}$ = 8.3425, *p* = .004, all *n* = 382). Adults that successfully hatched an egg were only marginally heavier (mean, 733 ± 69.8 g, *n* = 268) than those that failed (mean 724 ± 80.9 g, *n* = 110: LRT $\chi_{1}^{2}$ = 0.5745, *p* = .448, *n* = 378). There was also no evidence that body condition during the incubation period differed between the sexes (males: mean 736 ± 74.1 g, *n* = 178, females 727 ± 71.4 g, *n* = 174, LRT $\chi_{1}^{2}$ = 0.058, *p* = .810, *n* = 352) or between years they were monitored (LRT $\chi_{4}^{2}$ = 3.162, *p* = .531, *n* = 387).

The model that best described variation in body condition over the incubation period included the proportion of the incubation shift completed (incubation shift proportion), and the length of the incubation shift undertaken (incubation shift length), both as individual main effects and as an interactive effect ($R_{m}^{2}$ = 0.467, $R_{c}^{2}$ = 0.710, *n* = 387, Table 1). Incubation shift proportion significantly negatively affected body condition: Adults that had completed more than half of the shift were on average 99 g lighter (mean 664 ± 56 g, *n* = 117) than those still completing the first half of the shift (mean: 763 ± 57 g, *n* = 248). Likewise, incubation shift length significantly negatively affected body condition: Tropicbird undertaking long incubation shifts (7–13 days in length) were on average 16 g lighter (mean: 726 ± 77 g, *n* = 248) than those undertaking short incubation shifts (1–6 days in length, mean: 741 ± 63 g, *n* = 139). Tropicbirds entering long incubations shifts had higher body mass at the start of their incubation shift than tropicbirds entering short incubations shifts (Appendix S2, Figure S1a). Tropicbirds were much lighter by the end of long incubations shifts than at the end of short incubation shifts (Appendix S2, Figure S1b).

**TABLE 1:** Results of generalised linear mixed models (GLMMs) assessing which variables were associated with Red‐billed Tropicbird mass during incubation at St Helena, using individual identification (Ring ID) and nest identification (Nest ID) as random effects (top section) and parameter estimates of variables in the most parsimonious model (bottom section).

Competing models					
Model	Random effects	*k*	AICc	Delta AIC	AICcWt
(4) Incubation period days: Incubation shift length	Ring ID + Nest ID	7	3999.46	0.00	0.40
(3) Incubation days	Ring ID + Nest ID	8	3999.65	0.19	0.36
(2) Incubation shift proportion: Incubation period days	Ring ID + Nest ID	9	4001.10	1.64	0.18
(1) Global	Ring ID + Nest ID	10	4003.18	3.72	0.06

Significance of variables included in model (4)				
Variable	Estimate	SE	*t*	*p* Value
Incubation shift proportion	−0.063	0.003	−21.998	**<.001**
Incubation shift length	−0.008	0.004	−2.223	**.026**
Incubation shift proportion: Incubation shift length	−0.020	0.003	−7.155	**<.001**

*Note*: The global model contained all variables previously identified as significant predictors of body condition during incubation by LRTs: incubation shift length, incubation shift proportion and day of incubation period (incubation period days) included as additive and two‐way interaction effects. Other models denote the term that is removed from the global model and the resulting change in AICc. Model selection was based on AICc. *k* denotes the number of estimated parameters for each model. Akaike weights are represented as AICcWt.

Significant *p*‐values are in bold.

### 3.3 Incubation shifts and mass loss

**FIGURE 3:**
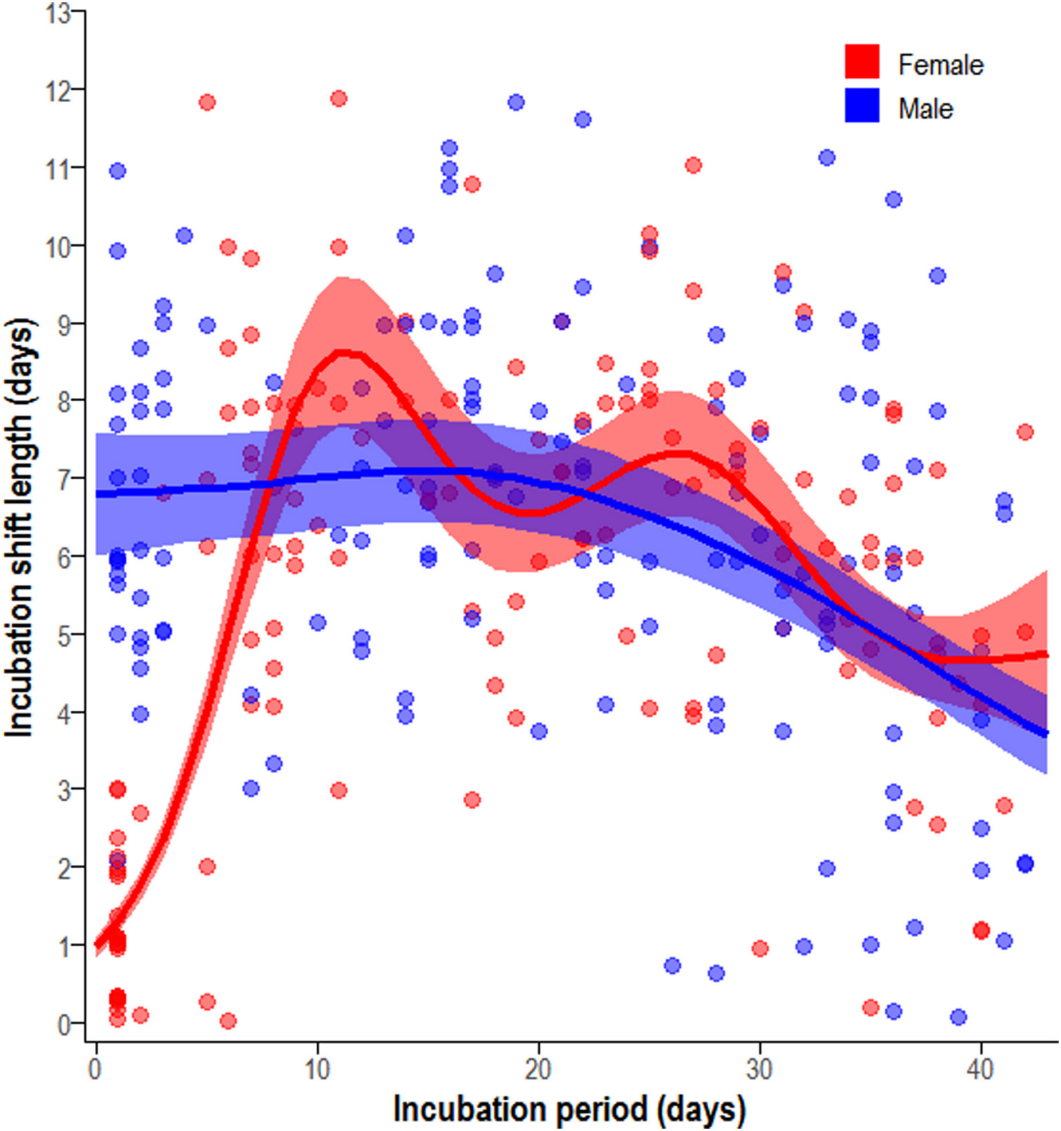
Variation in the incubation shift length (consecutive time spent incubating an egg) throughout the incubation period, in male (blue) and female (red) Red‐billed Tropicbirds at St Helena. Predicted values (line ±1 SD) are based on the final GAMM model (Table 3, bottom section), using 2013 as the reference year.

Red‐billed Tropicbird parents alternated incubating the egg in shifts, completing an average of 7.61 ± 1.20 shifts (range: 6–11 shifts, *n* = 23 nests) through the incubation period, with each shift lasting on average 6.04 ± 2.76 days (range < 1–12 days, *n* = 314: Appendix S2, Table S1). The model that best described variation in incubation shift length included an interaction between the day of the incubation period and sex, as well as incubation period, sex and year as main effects (Table 2). The incubation shift length was strongly influenced by parent sex, and by the day of the incubation period (Table 2 (bottom section), Figure 3). The first shift completed by the female after she had laid the egg was usually short (1.40 ± 1.44 days, Appendix S2, Table S1), and shift length of both sexes decreased as hatching approached (Figure 3, Appendix S2, Table S1). Males were more likely to complete longer incubation shifts than females (*β =* 0.269 ± 0.065 SE, *p* < .001, *t* ^1^ = 4.136), lasting on average 1 day longer (male mean shift length 6.44 ± 2.75 days, range 0.07–11.8, *n* = 148: females: 5.51 ± 2.94 days, range 0.01–11.9, *n* = 145). There was also considerable variation in shift length between years (*p* = .007, *F*_4_ = 3.616), incubation shifts during 2015 were on average the longest, lasting 7.00 ± 2.10 days, (range 0.94–11.8, *n* = 54), compared with the shortest shift lengths in 2016, lasting on average 5.15 ± 3.58 days (range 0.01–11.8, *n* = 46). Nesting attempts that successfully hatched an egg completed only marginally longer incubation shifts than those that did not (*p* = .185, *F*_1_ = 1.765, hatched: 6.07 ± 2.52 days, range 0.10–11.8, *n* = 229, failed 5.48 ± 3.67 days, range 0.01–11.9, *n* = 60).

**TABLE 2:** Generalised additive mixed models (GAMMs) assessing which variables were associated with incubation shift length of Red‐billed Tropicbirds at St Helena, using individual identification (Ring ID) and nest identification (Nest ID) as random effects (top section), and parameter estimates of variables in the most parsimonious model (bottom section).

Competing models				
Model	Random effects	AICc	BIC	LogLik
(3) Hatching success	Ring ID + Nest ID	1450.3	1497.9	−712.1
(2) Incubation days	Ring ID + Nest ID	1451.2	1502.5	−711.6
(1) Global	Ring ID + Nest ID	1453.2	1508.2	−711.6

Significance of smoothed terms in model 3			
Variable	edf	*F*	*p* Value
Incubation period days: Female	4.355	63.10	**<.001**
Incubation period days: Male	1.994	11.55	**<.001**

*Note*: The global model contained sex, hatching success and year as additive terms, the day of incubation period (Incubation period days) a smoothed additive term as well as an interaction between sex. Other models denote the term that is removed from the global model and the change in AICc this produced. Model selection is based on AICc.

Significant *p*‐values are in bold.

The proportion of an incubation shift completed by adult Red‐billed Tropicbirds was negatively related to the proportion of mass lost per day (LRT $\chi_{1}^{2}$ = 9.689, *p* = .002, *n* = 88), with an average of 4.1% (34 g), (range 2.7%–5.9%, 20–50 g, *n* = 6) of body mass loss per day within the first half of a shift compared with 2.7% (21 g) (range 0.4%–5.6%, 3–47 g, *n* = 79) of body mass lost per day in the last half of an incubation shift. There was no evidence that the proportion of mass loss per day differed between sexes, years, hatching outcome, incubation shift length or day of the incubation period (Sexes: LRT $\chi_{1}^{2}$ = 0.1892, *p* = .664, male: *n* = 40, female: *n* = 48, years: LRT $\chi_{4}^{2}$ = 1.330, *p* = .856, *n* = 88, incubation outcome: LRT $\chi_{1}^{2}$ = 0.080, *p* = .777, hatched: *n* = 50, failed: *n* = 38, incubation shift length LRT $\chi_{1}^{2}$ = 0.603, *p* = .437, *n* = 88, day of incubation period LRT $\chi_{1}^{2}$ = 0.199, *p* = .655, *n* = 81).

The model that best described the proportion of mass lost per day included the proportion of the shift completed and incubation shift length as additive effects ($R_{c}^{2}$ = .202, $R_{m}^{2}$ = .190, *n* = 81, Table 3 (top section), Appendix S2, Figure S2). Adults at the start of a shift lost a higher proportion of mass per day than those at the end of their incubation shift. There was also a marginal negative relationship between mass loss and the length of the shift being undertaken: The longer the shift the lower the proportion of mass loss per day (Table 3, bottom section).

**TABLE 3:** Generalised linear mixed models (GLMMs) assessing which variables were associated with Red‐billed Tropicbird proportion of mass loss per day during an incubation shift, using individual identification (Ring ID) and nest identification (Nest ID) as random effects (top section) and parameter estimates of variables in the most parsimonious model (bottom section).

Competing models					
Model	Random effects	*k*	AICc	Delta AIC	AICcWt
(4) Incubation period days	Ring ID + Nest ID	6	259.28	0.00	0.57
(3) Incubation shift Proportion: Incubation period days	Ring ID + Nest ID	7	260.80	1.52	0.27
(3) Incubation shift proportion: Incubation shift length	Ring ID + Nest ID	8	262.48	3.20	0.12
(2) Incubation shift length: Incubation period days	Ring ID + Nest ID	9	264.87	5.59	0.03
(1) Global	Ring ID + Nest ID	10	267.47	8.18	0.01

Significance of variables in model 4				
Variable	Estimate	SE	*t*	*p* Value
Incubation shift proportion	−0.160	0.047	−3.439	**<.001**
Incubation shift length	−0.093	0.050	−1.840	.066

*Note*: The global model contained all variables previously identified as significant predictors of body condition during incubation by likelihood ratio tests: (incubation shift length, proportion of incubation shift completed (Incubation shift proportion) and day of incubation period (Incubation period days)) included as additive and two‐way interactive effects. Other models denote the term that is removed from the global model and the change in AICc this produced. Model selection is based on AICc. *k* denotes the number of estimated parameters for each model. Akaike weights are represented as AICcWt.

Significant *p*‐values are in bold.

### 3.4 Parent attendance and body condition during chick rearing

**FIGURE 4:**
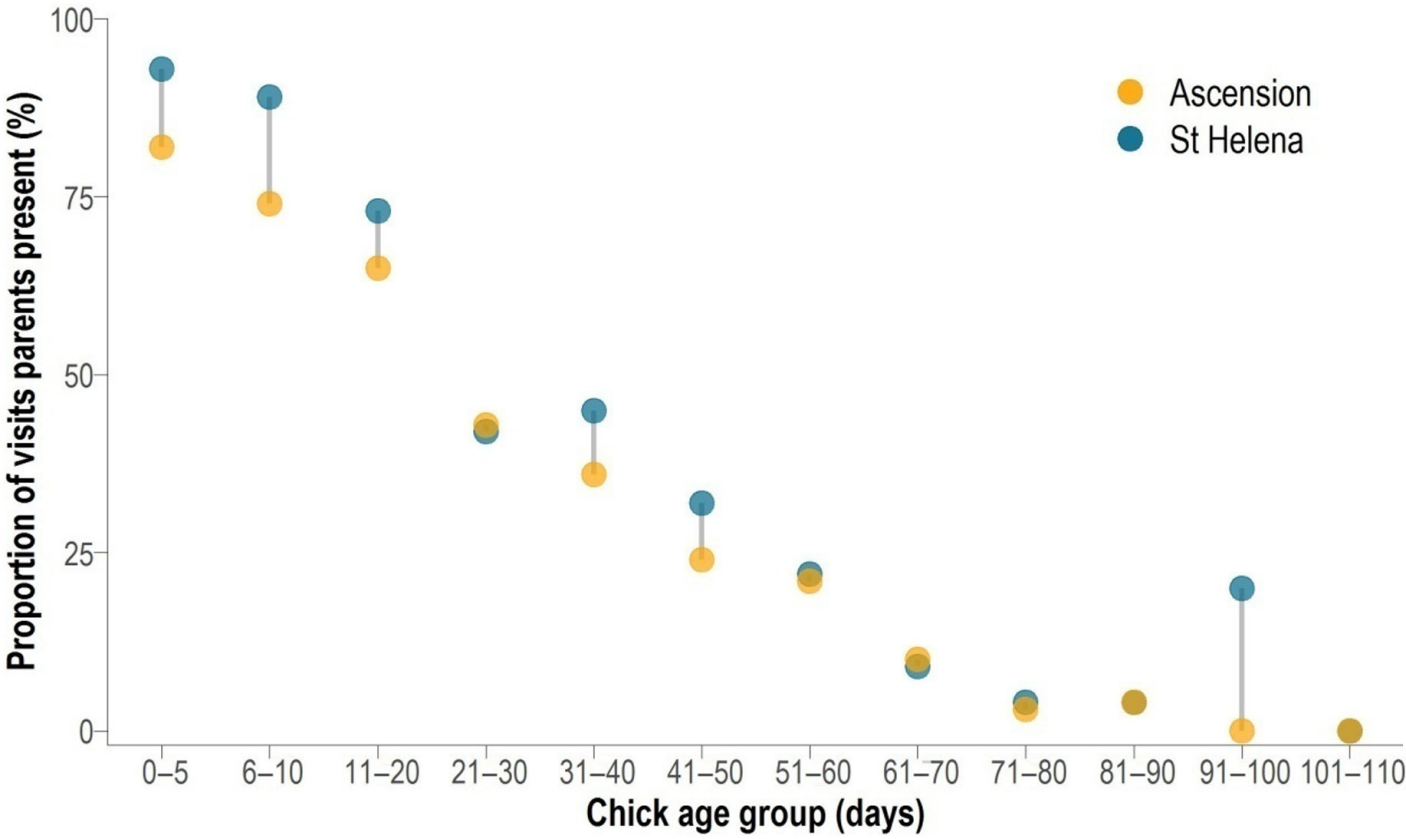
Relationship between the proportion of visits on which at least one parent was present, and chick age from two localities: St Helena 2013–2017 (blue) and Ascension 1957–1959 (yellow, from Stonehouse (1962)).

Red‐billed Tropicbird parents were found on the nest with the chick throughout its development until fledging, but parental attendance decreased substantially with chick age. Attendance was highest in the first 2 weeks of the chick's life, when parents were brooding and guarding the chick almost continuously, after which attendance reduced. Attendance during the fourth week of chick life showed the largest decrease, by almost 34% (Appendix S2, Table S2). Overall, adult Red‐billed Tropicbirds at St Helena were present with the chick on a significantly higher proportion of visits than those observed by Stonehouse (1962) on Ascension Island (*p* < .001, $\chi_{1}^{2}$ = 67.793, St Helena: 54.9%, Ascension: 35.7%; Figure 4), although the temporal pattern of attendance through the age groups were similar on both islands. On St Helena, there was also no relationship between the proportion of visits where at least one parent was present and the proportion of nests that successfully fledged a chick (*p* = .273, $\chi_{1}^{2}$ = 1.203, fledged: 70.1%, failed: 73.6%).

After accounting for repeated measures of individuals and nests, there was no significant difference in body condition between days during of the chick rearing phase (LRT: $\chi_{1}^{2}$ = 0.023, *p* = .880, *n* = 290); however, body condition did vary significantly between weeks, notably adults rearing chicks at 4 weeks of age were likely to be lighter (*β* = −0.057 ± 0.019, *t* = −2.953, *p* = .003), averaging 26 g less (mean 677 ± 42 g, *n* = 23) than those rearing chicks in the first week of life (mean 703 ± 59 g, *n* = 73, Appendix S2, Figure S3). There was no significant difference in body condition between the sexes (LRT: $\chi_{1}^{2}$ = 0.157, *p* = .692, male: *n* = 140, females: *n* = 150), between years (LRT: $\chi_{4}^{2}$ = 1.606, *p* = .808, *n* = 290) or between adults that successfully fledged a chick and those that did not (LRT: $\chi_{1}^{2}$ = 0.911, *p* = .240, fledged: *n* = 178, failed: *n* = 112).

### 3.5 Chick growth

**FIGURE 5:**
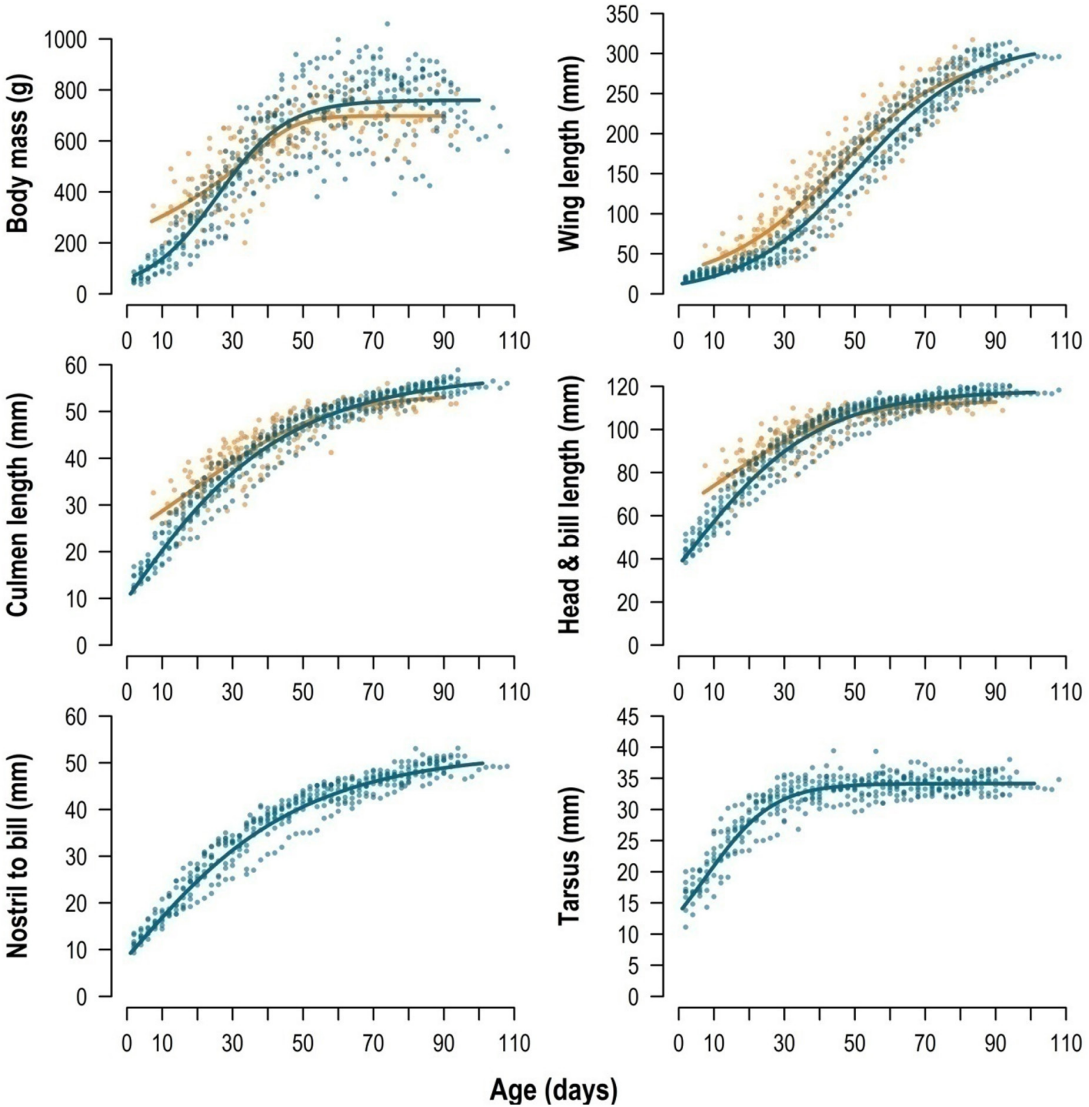
Growth rates of Red‐billed Tropicbird chicks during the 2017 breeding season from two geographical regions: St Helena, *n* = 12 chicks: blue lines/circles and Cabo Verde, *n* = 22 chicks, orange lines circles. Lines are based on logistic growth curves (see Table 4 for parameter estimates). Nostril to bill and tarsus were not measured on Cabo Verde chicks. Body mass at both locations took approximately 60 days reach an asymptote and wing length starting to increase strongly after about 20 days when primary feathers started to grow. Structural variables relating to skeletal growth such as culmen length, head and bill length and nostril to bill at St Helena appeared to take about 60–80 days to reach an asymptote, whereas tarsus took approximately 40 days to reach asymptote on St Helena.

On St Helena, 12 chicks were monitored from two up to 108 days after hatching, including eight chicks until fledging. At Cabo Verde, 22 chicks were monitored from a minimum of 7 days up to a maximum of 93 days after hatching, including seven chicks that were monitored until fledging. Growth curves were very similar across the two locations (Figure 5). Chicks from St Helena were heavier: The estimated asymptote of body mass was 760 g at St Helena and 695 g at Cabo Verde (Table 4). Body mass also showed larger fluctuations on St Helena, with chicks increasing up to 25% of their body mass per day, compared with only 5% at Cabo Verde. The maximum daily increase in body mass at both locations was recorded when the chicks were between 20 and 30 days old: during this period some chicks increased their mass by up to 47% (336 g) per day at St Helena, and 17% per day (increase of up to 270 g/day) at Cabo Verde. Mean daily chick growth rates across the chick rearing period at St Helena were 3.6% of body mass and 2.1% of body mass at Cabo Verde (increase of 6.6 and 7.4 g/day, respectively, assuming linear growth: for logistic growth curve parameters see Table 4). St Helena chicks gained significantly higher proportions of body mass per day than chicks from Cabo Verde (estimate 0.02%, *t* = 2.454, *p* = .032, Appendix S2, Figure S4). A maximum body mass of 1059 g was reached after 74 days at St Helena and 840 g after 63 days at Cabo Verde.

**TABLE 4:** Parameter estimates for logistic growth curves of Red‐billed Tropicbird chicks in 2017 measured between age 2 and 108 days on St Helena (*n* = 12) and between 11 and 93 days of age (*n* = 22) for Cabo Verde.

St Helena						Cabo Verde			
Variable	Parameter	Estimate	SE	*t*	*p*	Estimate	SE	*t*	*p*
Weight	Asymptote	760.1	11.1	68.3	.000	698.1	13.6	51.3	.000
	*m*	1.1	0.5	2.1	.039	9.3	7.5	1.2	.215
	*k*	0.1	0.0	5.3	.000	0.2	0.1	1.5	.136
	Inflexion	25.9	2.4	11.0	.000	35.5	6.3	5.7	.000
Wing	Asymptote	314.2	8.1	38.7	.000	294.7	18.5	15.9	.000
	*m*	0.9	0.2	3.9	.000	1.6	1.0	1.7	.095
	*k*	0.1	0.0	8.5	.000	0.1	0.0	2.9	.004
	Inflexion	49.6	1.2	40.0	.000	46.5	3.5	13.4	.000
Culmen	Asymptote	58.2	0.8	74.1	.000	53.7	1.5	36.1	.000
	*m*	−0.6	0.2	−3.0	.003	2.9	2.6	1.1	.266
	*k*	0.0	0.0	10.4	.000	0.1	0.0	2.5	.013
	Inflexion	1.8	3.3	0.6	.575	19.1	10.5	1.8	.071
Head and bill	Asymptote	118.2	0.9	134.9	.000	113.3	1.8	61.4	.000
	*m*	0.2	0.4	0.5	.631	3.4	3.6	1.0	.340
	*k*	0.1	0.0	10.4	.000	0.1	0.0	2.6	.010
	Inflexion	3.8	3.0	1.3	.198	9.5	12.2	0.8	.438
Tarsus	Asymptote	34.2	0.2	222.4	.000				
	*m*	1.8	0.9	2.1	.040				
	*k*	0.1	0.0	7.1	.000				
	Inflexion	6.8	2.6	2.6	.009				
Nostril to bill	Asymptote	52.3	1.0	53.5	.000				
	*m*	−0.5	0.2	−2.3	.021				
	*k*	0.0	0.0	8.4	.000				
	Inflexion	3.9	4.0	1.0	.329				

Maximum body mass of chicks from both locations was considerably greater than that of adults: at St Helena 147% of mean adult weight (721 g, A. Beard, personal observation) and at Cabo Verde 133% of mean adult weight (631 g, S. Saldanha, personal communication). Of the chicks that were monitored until fledging, body mass declined with increasing age: St Helena chicks lost on average 17 g in the 10 days prior to fledging, and Cabo Verde chicks lost on average 25 g over the same period (average decrease of 0.008%, *n* = 8 and 0.006%, *n* = 7 of body mass per day respectively). Although the final mass measured prior to fledging at St Helena (mean: 681 g) was on average 5% lower than mean adult body mass at St Helena (mean: 721 g), this apparent difference was not significant (*t* = −1.81, df = 7.12, *p* = .113). At Cabo Verde, chicks were 2.7% heavier at fledging (mean: 648 g) than the average adult. Chicks of fledging age from St Helena were also structurally smaller than an average adult: wing 6%, head and bill 2%, feathers to bill and nostril to bill were 8% smaller than the average adult reported by (Nunes et al., 2013) from the Abrolhos archipelago off the coast of Brazil, but bigger than chicks at fledging from Cabo Verde: wing 6%, head and bill 4%, feathers to bill 8% bigger.

## 4 DISCUSSION

This study is the first to provide a comprehensive examination of body condition influences on the breeding regime of a long‐lived pelagic species providing bi‐parental care. We found clear differences in adult body condition between years and life stages. The flexible sex‐specific incubation routines, where males undertook longer and more frequent incubation shifts than females, were unexpected given that the species is considered monomorphic (Nunes et al., 2013). We identified key aspects of the species breeding ecology such as incubation regime and chick growth, filling knowledge gaps essential for the effective conservation and management of this globally threatened species.

### 4.1 Between‐year variation in adult body condition at St Helena

Adult red‐billed Tropicbirds showed considerable year‐to‐year variation in body condition in our 2013–2017 study, with birds being significantly lighter during 2015 when adults also conducted significantly longer incubation shifts. This co‐variation in adult body condition and incubation shift length suggests substantial inter‐annual fluctuations in prey availability, with incubating adults in 2015 having to wait for longer periods for their partner to return from foraging at sea. Our study was conducted in part, during the austral winter (July–September) which is considered the peak in the Angola Benguela upwelling cycle (Hagen et al., 2005). St Helena is directly in the path of the south‐east trade winds which drives the South Equatorial Current, bringing relatively enriched waters from the Angola Benguela upwelling current system towards the island. However, while there were no extreme coastal warm or cold anomalous events during the study period that would be classified as a Benguela Niño or Niña (Imbol et al., 2021; Lübbecke et al., 2019), a moderate cold coastal event along the Angolan‐Namibian coastlines did occur during 2015 (Imbol Koungue et al., 2019). It is possible that the inter‐annual fluctuations observed by this study, particularly during 2015, may be a consequence of variation in oceanographic conditions known to occur in the south‐east Atlantic Ocean resulting from variability in the Benguela current upwelling system.

Environmental pressures and availability of resources may vary at different times of year, this may in turn also effect body condition and breeding regime. For example Red‐tailed Tropicbirds (*Phaethon rubricauda*) on Round Island, Mauritius, which like Red‐billed Tropicbirds at St Helena, breed year‐round but with a degree of seasonality, were shown to have different flight efforts and costs in summer and winter (Shepard, 2021). This study only conducted fieldwork during the peak of the populations year‐round breeding season (August to December) and although body condition was not significantly associated with date (Julian day) within this peak period, studying Red‐billed Tropicbirds breeding across the whole of the annual cycle would offer further insights into body condition regulation.

Many seabirds, including some tropicbird species, show a marked seasonality in breeding phenology, the onset of which is planned to coincide with the change of upwelling and marine productivity (Castillo‐Guerrero et al., 2011). This is to ensure that the period of highest food demand during the chick rearing period is well‐matched to the peak in food availability and enabling adult body condition to be maintained despite the demands of chick provisioning (Lack, 1968). Tropicbirds at St Helena showed no significant year‐to‐year variation in body condition across the incubation and chick rearing period, suggesting that availability of prey early in the peak season may be high and relatively stable. In times of food shortage, birds can generally respond by reducing clutch and/or egg size to reduce the required energy intake (Birkhead & Nettleship, 1982; Drent & Daan, 1980), however as tropicbirds lay a single egg, they may instead respond by not breeding, or by delaying breeding until environmental conditions are more favourable. Our findings suggest that there may be a body condition threshold when deciding to breed; adults may need to reach a certain body condition before initiating a breeding attempt, to balance the cost of reproductive effort against an individual's long‐term survival. This is further supported by the increase in body condition prior to breeding as a buffer against loss of body reserves across the breeding attempt, a common strategy in pelagic seabirds (Cherel et al., 1993).

### 4.2 Reproductive performance

This study found no relationship between reproductive performance and body condition (a) during the incubation period, (b) between incubation shifts, (despite their variable duration, ranging from <1 to 12 days) and (c) during the chick rearing period. There was also no evidence that parental attendance during chick rearing influenced fledging outcome, supporting the findings by Beard et al. (2023) during the same study period on St Helena, that significant between‐year variation in fledging success was driven by large between‐year variations in daily chick survival due to high levels of chick predation. Indeed, our study indicates that Red‐billed Tropicbirds can sustain body condition sufficiently to maintain their reproductive effort.

### 4.3 Sex‐specific incubation routine

This is the first study to describe significant sex differences in incubation routine in Red‐billed Tropicbirds, although it is not uncommon in other seabirds (Chaurand & Weimerskirch, 1994; Phillips, 1987; Pinet et al., 2012; Weimerskirch, 1995). Such sex differences reflect different reproductive roles, the male takes the first long incubation shift so that the female can recuperate body condition after laying the egg. As a consequence, males tend to have longer incubation shifts on average than females. Subsequently (after the first shift) incubation shifts changed in duration over the incubation period in a similar way in both males and females. The shorter incubation shifts observed closer to hatching suggests that Red‐billed Tropicbirds adjust the length of time spent at sea foraging and therefore the incubation shift length, to ensure that when the chick hatches the attending adult can feed the chick before its yolk reserve runs out. This foraging trip regulation strategy is common in pelagic seabirds with extended incubation periods and long incubation shifts (Gaston et al., 2014; Johnstone & Davis, 1990; Nam et al., 2008; Weimerskirch et al., 1986) and there are three mutually non‐exclusive hypotheses proposed to explain what could trigger this reduction in incubation shift length during the end of the incubation period: (1) endogenous timing, where adults can predict the timing of hatching, (2) response to embryo signals such as heart beat, or bill tapping providing a cue for parents to assess embryo development and (3) that the declining incubation shift durations towards hatching is a consequence of a seasonal increase in food availability (González‐Solís, 2004). Our data did not allow us to elucidate the underlying mechanisms for the reduction in incubation shift lengths close to hatching. It was also not possible to evaluate the level of synchrony within pairs during the incubation routine as not all incubation shifts for each pair and each nest could be identified. However, if we exclude the first shift, the results show similar consecutive shift lengths for the two sexes, suggesting a level of synchrony. Given that there was little evidence of differences in body condition between Red‐billed Tropicbird sexes through the incubation and chick rearing periods or in the proportion of mass lost per day during incubation, females and males likely have similar reproductive efforts and resulting impacts on their body condition.

### 4.4 Body condition regulation

The significant decrease in body condition across the incubation period seems to be in part mitigated by the higher body condition observed in pre‐breeding adults, suggesting that improved pre‐breeding body condition may help to meet the demands of breeding such as laying the egg in females, ensuring there are sufficient body reserves to fast during incubation and provisioning of food to the growing chick. The finding that adults with body greater condition are also able to undertake longer periods of fasting while incubating supports this hypothesis. Body condition generally decreased through incubation shifts, which adults presumably then attempt to replenish on the subsequent foraging trip. However, this is not always possible in seabirds, for example in male and female Streaked Shearwaters (*Calonectris leucomelas*) despite having similar mean incubation shift lengths, only male body condition decreased throughout the incubation period which was attributed to subsequent foraging trip performance (Nam et al., 2008). Given the observed overall negative effect of the incubation period on body condition in both sexes, adults in our study may not be completely successful in regaining body condition, also indicating that both sexes may have similar foraging behaviour in subsequent foraging trips.

This study shows that Red‐billed Tropicbirds can regulate energy expenditure (mass loss) regardless of the length of shift. Some seabird species are known to exhibit an initial drop in the rate of mass loss during the beginning of an incubation shift due to the stomach contents from the last meal being rapidly digested during the first few days of a fast (Weimerskirch, 1990) In other species, the daily mass loss can vary throughout a fast, for example Emperor Penguins (*Aptenodytes forsteri*) (Groscolas, 1988). Red‐billed Tropicbirds at St Helena show a constant rate of mass loss through incubation shifts in both sexes, the amount of which is influenced by how much of the incubation shift has been completed. Although the incubation shift length and incubation period did not significantly affect the proportion of mass lost per day, they were likely to have some effect overall as they were included in the final model. This model indicates that the further adults are into the incubation period, the rate of mass loss increases, suggesting that a Red‐billed Tropicbird's ability to regulate body condition decreases during the incubation period.

### 4.5 Parental attendance during the chick rearing period

The high adult attendance rate identified during the first 2 weeks of life when adults brood and guard the chick are consistent with similar findings in Red‐billed Tropicbirds on nearby Ascension Island (Stonehouse, 1962) and Red‐tailed Tropicbirds in the Indian Ocean (Diamond, 1975; Fleet, 1974). The observed differences in parental attendance of the chick between Ascension and St Helena may be a consequence of the different time periods studied (Figure 4). There may also be different environmental influences on attendance due to the different geographical regions: Ascension is closer to the equator and generally warmer (Ascension Island sea surface temperature: range 24 to 28°C (Hughes, 2013), St Helena: 20 to 26°C (Cowburn et al., 2021)) and therefore on Ascension parents may not need to brood over chick for as long to ensure adequate thermoregulation of the chick in the nesting cavity. Inter‐specific competition with other seabird species, particularly White‐tailed Tropicbirds (*Phaethon lepturus*) (Stonehouse, 1962) at Ascension may also cause parents to be less attentive to chicks overall than St Helena where Red‐billed Tropicbirds only have to deal with intraspecific competition (Annalea Beard, personal observation). If we consider parental attendance up to a nesting age of 50 days, then attendance of parents on Ascension (53%), Stonehouse (1962) and St Helena (66%) are similar, compared with the much higher attendance of 83% observed in the Galapagos in 1965–67 (Harris, 1969).

### 4.6 Chick growth

The Red‐billed Tropicbird growth rate of 6.6 g/day from St Helena and 8.4 g/day at Cabo Verde described here provides a baseline for comparison for future studies. The maximum mean chick mass observed on St Helena appears to be very similar to values previously reported from nearby Ascension Island (maximum mean mass 780 g: Stonehouse, 1962), but larger than on Cabo Verde during the same season. It is not uncommon for tropicbird chick maximum mass, prior to fledging to be greater than that of adults (Diamond, 1975; Phillips, 1987), or for chicks to lose mass prior to fledging (Stonehouse, 1962). The observed large daily fluctuations in chick mass are likely due to adult attendance and provisioning effort, particularly for one chick from St Helena which showed periodic weight declines where presumably its parents were limiting provisioning frequency and amount. Such fluctuations were not as apparent at Cabo Verde, possibly due to the difference in frequency of data collection (every ≤5 days, as opposed to 2 days at St Helena) and limited monitoring during the first 2 weeks after hatching, when the highest rates of chick growth occur. As with many seabirds, tropicbirds are known to adjust chick food provisioning depending on food availability (Ramos & Pacheco, 2003), which can in turn affect development (Schaffner, 1990) and ultimately chick survival. Despite similar growth rates between regions, the fledglings in our study were heavier and structurally bigger at St Helena than at Cabo Verde (Table 4, Figure 5), likely representing a geographical variation in body size among Atlantic populations of Red‐billed Tropicbirds, as shown for other tropicbird populations (Le Corre & Jouventin, 1999). These results agree with previous suggestions that phenotypic variation in adult body size among Red‐billed Tropicbird colonies in the Pacific Ocean are primarily driven by phenotypic plasticity and responses to local environmental conditions, rather than genetic variation among colonies (Piña‐Ortiz et al., 2022).

Only one of the 12 St Helena chicks studied in 2017 failed to fledge. The failure cause was due to predation rather than poor development or attendance related reasons, therefore due to their success, the growth rates identified are likely to be at the higher end of the range, and we speculate that food availability may have been relatively stable in 2017 for this to occur. Inter‐specific competition between seabird species may also be a factor affecting chick growth at Cabo Verde: two of the chicks in this study were recorded as being attacked by Cabo Verde Shearwaters (*Calonectris edwardsii*) which compete for nesting burrows with Red‐billed Tropicbirds on Raso Islet (S. Saldanha, personal communication). The level of post‐fledging care provided by Red‐billed Tropicbirds remains relatively little studied, although Ainley et al. (1986) suggested that parental care in tropicbirds may extend beyond departure from the nesting colony. The chicks that we studied on St Helena fledged with a final body mass not significantly smaller than adult mean body mass. This implies that chicks may have fledged with little to no food reserves, suggesting that they fledged before reaching mean adult size. It is advantageous for Red‐billed Tropicbirds to provide post‐fledging care, given its positive relationship with fledging survival probability (López‐Idiáquez et al., 2018).

## 5 CONCLUSIONS

This study identified between‐year differences in body condition and impacts of these differences on the incubation regime of Red‐billed Tropicbirds at St Helena. Although our study did not formally test for such an association, our findings are the first to indicate an apparent influence of the Angola Benguela upwelling system and the South Equatorial Current on reproduction and foraging behaviour of seabirds in the South Atlantic, although possibly less severe than the dramatic effects on seabird survival and reproduction documented for El Niño and decadal oceanographic oscillations (see Champagnon et al., 2018). These results highlight the importance of multi‐year studies and the need for further exploration of the impact of the Angola Benguela upwelling cycle on species and ecosystems.

Our results also provide new insights into the regulation of body condition across the breeding cycle in a pelagic seabird in the South Atlantic. In our study, Red‐billed Tropicbirds at St Helena demonstrated flexible sex‐specific incubation regimes that were related to reproductive role. Despite variation in body condition in some years, reproductive performance was not affected, indicating that Red‐billed Tropicbirds are able to mitigate loss of condition in years when food availability is likely to be relatively low. The flexibility and adaptability of body condition regulation in this pelagic species described here will be particularly important for threatened populations to optimise resources as global climate change and its effects on the marine environment influence prey availability.

## AUTHOR CONTRIBUTIONS

**Annalea Beard:** Conceptualization (lead); data curation (supporting); formal analysis (lead); funding acquisition (supporting); investigation (lead); methodology (supporting); project administration (lead); visualization (supporting); writing – original draft (lead); writing – review and editing (lead). **Robert J. Thomas:** Formal analysis (supporting); investigation (supporting); methodology (supporting); supervision (supporting); writing – original draft (supporting). **Renata Medeiros Mirra:** Formal analysis (supporting); investigation (supporting); methodology (supporting); writing – original draft (supporting). **Elizabeth Clingham:** Data curation (supporting); project administration (supporting). **Leeann Henry:** Data curation (supporting); investigation (supporting). **Sarah Saldanha:** Data curation (supporting); investigation (supporting); resources (supporting). **Jacob González‐Solís:** Supervision (supporting); writing – original draft (supporting). **Frank Hailer:** Formal analysis (supporting); investigation (supporting); methodology (supporting); supervision (supporting); writing – original draft (lead).

## Supporting information


Data S1.

Data S2.

Data S3.

Data S4.

Data S5.

Data S6.

Data S7.

Data S8.

Data S9.

Appendix S1 and S2.

## Data Availability

All data and code are provided as [Link], [Link], [Link], [Link], [Link], [Link], [Link], [Link], [Link], [Link].
