# Supplementary material for: Between‐year and spatial variation in body condition across the breeding cycle in a pelagic seabird, the Red‐billed Tropicbird
Source: Ecol Evol. 2023 Dec 27;13(12):e10743. doi: 10.1002/ece3.10743 (PMC10752250; doi:10.1002/ece3.10743)
Supplement: Supplementary file 5 — Data S5. [file ECE3-13-e10743-s009.docx]

##~~~~~~~~~~~~~~~~~~~~~~~~~~~~~~~~~~~~~~~~~~~~~~~~~~~~~~~~~~~~~~~~~~~~~~~~~~~~~~

## --

##----------------- RED-BILLED TROPICBIRD ADULT MASS ANALYSIS-------------------

## --

##~~~~~~~~~~~~~~~~~~~~~~~~~~~~~~~~~~~~~~~~~~~~~~~~~~~~~~~~~~~~~~~~~~~~~~~~~~~~~~

# FOR SUBMISSION ALONGSIDE THE MANUSCRIPT ENTITLED "Among-year and spatial

#variation in body condition across the breeding cycle in a pelagic seabird, the Red-billed Tropicbird"

#SUBMITTED TO ECOLOGY AND EVOLUTION JOURNAL FOR CONSIDERATION JULY 2023

#last updated 04/07/2023

#R studieo version 1.2.5019

##~~~~~~~~~~~~~~~~~~~~~~~~~~~~~~~~~~~~~~~~~~~~~~~~~~~~~~~~~~~~~~~~~~~~~~~~~~~~~~

## INSTALL PACKAGES ----

##~~~~~~~~~~~~~~~~~~~~~~~~~~~~~~~~~~~~~~~~~~~~~~~~~~~~~~~~~~~~~~~~~~~~~~~~~~~~~~

library(lubridate)

library(lme4)

library(mgcv)

library(gamm4)

library(MuMIn)#for calculating r2 and r2c values in glmm's

library(hrbrthemes)# for standardising plots in ggplot

library(ARTofR)# for standardising script structure

library(ggplot2)

library(tidyverse)

library(lemon)# for axis manipulation in ggplot2

library(lmtest)

library(AICcmodavg)

library(data.table)

library(viridis)

library(ggpubr)# FOR COMBINING MULTIPLE PLOTS

##~~~~~~~~~~~~~~~~~~~~~~~~~~~~~~~~~~~~~~~~~~~~~~~~~~~~~~~~~~~~~~~~~~~~~~~~~~~~~~

## STATISTICS: ADULT BODY CONDITION ----

##~~~~~~~~~~~~~~~~~~~~~~~~~~~~~~~~~~~~~~~~~~~~~~~~~~~~~~~~~~~~~~~~~~~~~~~~~~~~~~

#............................LOAD DATA...........................####

#SET THE WORKING DIRECTORY

#setwd("C:\\Users\\X")

#READ THE CSV FILES

dframe1 <- read.csv("ADULT_MASS.csv") # Read in the adult weights data

#..........................FORMAT DATA ..........................####

# Extract date values

date <- parse_date_time(dframe1$Date, "dmy")

date

# Year as a number

dframe1$year <- year(date)

dframe1$year

# Year as a factor

dframe1$Fyear <- as.factor(dframe1$year)

# ordering the Activity categories in a temporal sequence

dframe1$Activity2 <- factor(dframe1$Activity, levels=c("NONBREEDING", "PREBREEDING", "INCUBATING","CHICK REARING"))

summary(dframe1$Activity2)# NONBREEDING=278, PREBREEDING=31, INC=415, CR=337

dframe1$Fyear <- factor(dframe1$Fyear, levels=c("2013", "2014","2015","2016","2017"))

##~~~~~~~~~~~~~~~~~~~~~~~~~~~~~~~~~~~~~~~~~~~~~~~~~~~~~~~~~~~~~~~~~~~~~~~~~~~~~~

## SIMPLE SUMMARIES ----

##~~~~~~~~~~~~~~~~~~~~~~~~~~~~~~~~~~~~~~~~~~~~~~~~~~~~~~~~~~~~~~~~~~~~~~~~~~~~~~

#differences in mass by sex

output1<- dframe1 %>% mutate(count=1) %>% group_by(Sex) %>%

summarise(n=sum(count), meanw=mean(Weight),Weightsd=sd(Weight),wmin=min(Weight), wmax=max(Weight))

output1

#differences in mass by breeding stage

output2<- dframe1 %>% mutate(count=1) %>% group_by(Activity2) %>%

summarise(n=sum(count), meanw=mean(Weight),Weightsd=sd(Weight),wmin=min(Weight), wmax=max(Weight))

output2

#differences in mass by year

output3<- dframe1 %>% mutate(count=1) %>% group_by(Fyear) %>%

summarise(n=sum(count), meanw=mean(Weight),Weightsd=sd(Weight),wmin=min(Weight), wmax=max(Weight))

output3

##~~~~~~~~~~~~~~~~~~~~~~~~~~~~~~~~~~~~~~~~~~~~~~~~~~~~~~~~~~~~~~~~~~~~~~~~~~~~~~

## FIGURE 2 MASS V STAGE ----

##~~~~~~~~~~~~~~~~~~~~~~~~~~~~~~~~~~~~~~~~~~~~~~~~~~~~~~~~~~~~~~~~~~~~~~~~~~~~~~

# plot of adult body condition (mass in g) of adult red-billed tropicbirds at different breeding stages

dframe1$Activity2 <- factor(dframe1$Activity2, levels=c("CHICK REARING","INCUBATING","PREBREEDING","NONBREEDING"))

ggplot(dframe1, aes(x=Activity2, y=Weight))+

geom_boxplot(fill="lightgrey", notch = TRUE,notchwidth = 0.5)+

scale_y_continuous(limits=c(500,1000), expand=c(0, 0.005))+

scale_x_discrete(labels=c("Chick \nrearing ","Incubating ","Pre- \nbreeding ","Non- \nbreeding "))+

stat_summary(fun.y=mean, geom="point", shape=21, size=3, color="black", fill="white") +#add mean of each year as point

labs(x="Breeding stage", y="Adult mass (g)")+

geom_text(data=output2,aes(Activity2, Inf, label=n), vjust=0.5,hjust=1, size=3.5)+

coord_flex_flip(left=brackets_vertical(length=0.07,direction="right"), bottom=capped_horizontal("none"))+

theme_ipsum(grid=FALSE, axis=TRUE,

axis_title_size = 14,

base_size = 12,

axis_title_face = "bold",

axis_title_just = "mc",

axis_col = "black",

ticks=TRUE)+

theme(plot.margin = unit(c(1, 1, 1, 1), "cm"))

##~~~~~~~~~~~~~~~~~~~~~~~~~~~~~~~~~~~~~~~~~~~~~~~~~~~~~~~~~~~~~~~~~~~~~~~~~~~~~~

## FIGURE 3 MASS VERSUS YEAR ----

##~~~~~~~~~~~~~~~~~~~~~~~~~~~~~~~~~~~~~~~~~~~~~~~~~~~~~~~~~~~~~~~~~~~~~~~~~~~~~~

ggplot(dframe1, aes(x=Fyear, y=Weight))+

geom_violin(width=0.8,alpha=0.75,fill="grey", color="grey")+

geom_boxplot(width=0.4, alpha=1, colour="black",fill="white",outlier.colour="black", outlier.size=3,outlier.fill="black")+#notch=TRUE,aes(colour=Activity2))+

stat_summary(fun.y=mean, geom="point", shape=21, size=3, color="black", fill="white") +#add mean of each year as point

scale_y_continuous(limits=c(500,1000), expand=c(0, 0.005))+

scale_x_discrete(labels=c("2013","2014","2015","2016","2017"))+

geom_text(data=output3,aes(Fyear, Inf, label=n), colour="black", vjust=1,hjust=.5, size=3.5)+

labs(x="Year", y="Adult mass (g)")+

theme_ipsum(grid=FALSE, axis=TRUE,

axis_title_size = 14,

base_size = 12,

axis_title_face = "bold",

axis_title_just = "mc",

axis_col = "black",

ticks=TRUE)+

theme(legend.position="none")

##~~~~~~~~~~~~~~~~~~~~~~~~~~~~~~~~~~~~~~~~~~~~~~~~~~~~~~~~~~~~~~~~~~~~~~~~~~~~~~

## STATISTICS: INFLUENCES ON ADULT BODY CONDITION ----

##~~~~~~~~~~~~~~~~~~~~~~~~~~~~~~~~~~~~~~~~~~~~~~~~~~~~~~~~~~~~~~~~~~~~~~~~~~~~~~

#effect of julian day

#DOES BODY CONDITION VARY SIGNIFICANTLY WITHIN THE BREEDING SEASON?

model1 <- gamm(Weight ~ s(Julian.day, k=-1), random= list(Ring_Nr = ~1, Waypoint= ~1),

family=Gamma(link="log"), data=dframe1)

summary(model1$gam)

# julian day,edf= 1, ref.df= 1, f=0.411, p= 0.521, R-sq.(adj) = -0.00169, n = 1061

#generalised linear models testing between year differences in adult body condition

#not used in the manuscript

#m0<- glmer(Weight ~ 1+ (1|Ring_Nr) +(1|Waypoint),family=Gamma(link="log"),data=dframe1, na.action=na.exclude)

#m1<- glmer(Weight ~ Fyear+ (1|Ring_Nr) +(1|Waypoint),family=Gamma(link="log"),data=dframe1, na.action=na.exclude)

#lrtest(m0, m1)

# YEAR # chisqu= 18.759 0.0008763 *** df=4, n=1061

#significant between year differences in body condition

#final model accounting for differences in breeding stage between years

m0a<- glmer(Weight ~ Activity2+ (1|Ring_Nr) +(1|Waypoint),family=gaussian(link="log"),data=dframe1, na.action=na.exclude)

m1a<- glmer(Weight ~ Activity2+Fyear+ (1|Ring_Nr) +(1|Waypoint),family=gaussian(link="log"),data=dframe1, na.action=na.exclude)

lrtest(m0a, m1a)

#df=4 chisq=11.261 p= 0.02378 *, n=1061

#after accounting for differences in breeding stages there are still significant between year differences in body condition

#general linear models testing for differences between breeding stages, sexes, sex x breeding stages

m0<- glmer(Weight ~ 1+ (1|Ring_Nr) +(1|Waypoint),family=Gamma(link="log"),data=dframe1, na.action=na.exclude)

m1<- glmer(Weight ~ Activity2 + (1|Ring_Nr) +(1|Waypoint),family=Gamma(link="log"),data=dframe1, na.action=na.exclude)

m2<- glmer(Weight ~ Sex+ (1|Ring_Nr) +(1|Waypoint),family=Gamma(link="log"),data=dframe1, na.action=na.exclude)

m3<- glmer(Weight ~ Sex+ Activity2+Sex: Activity2+ (1|Ring_Nr) +(1|Waypoint),family=Gamma(link="log"),data=dframe1, na.action=na.exclude)

m4<- glmer(Weight ~ Activity2+Sex+ (1|Ring_Nr) +(1|Waypoint),family=Gamma(link="log"),data=dframe1, na.action=na.exclude)

lrtest(m0, m1) # significant differences in condition during different breeding stages

# ACTIVITY #CHISQ=76.888 P= 2.2e-16 ***, df3, N=1061

lrtest(m0, m2) # no significant difference between the sexes

# SEX # CHISQ=0.0072 P= 0.9326, df=1

lrtest(m4, m3)#no significant difference in body condition between the sexes during different breeding stages

#ACTIVITY X SEX INTERACTION # CHISQ=3.2187 P= 0.3591, df=3

##~~~~~~~~~~~~~~~~~~~~~~~~~~~~~~~~~~~~~~~~~~~~~~~~~~~~~~~~~~~~~~~~~~~~~~~~~~~~~~

## STATISTICS: MASS LOSS DURING INCUBATION ----

##~~~~~~~~~~~~~~~~~~~~~~~~~~~~~~~~~~~~~~~~~~~~~~~~~~~~~~~~~~~~~~~~~~~~~~~~~~~~~~

#............................LOAD DATA...........................####

#SET THE WORKING DIRECTORY

#setwd("C:\\Users\\X")

#READ THE CSV FILE

dframe2 <- read.csv("ADULT_MASS_DURING_INCUBATION.csv")# 389 OBS

#..........................FORMAT DATA ..........................####

# Extract date values

date <- parse_date_time(dframe2$Date, "dmy")

date

# Year as a number

dframe2$year <- year(date)

dframe2$year

# Year as a factor

dframe2$Fyear <- as.factor(dframe2$year)

summary(dframe2$Fyear) # request sample sizes

dframe2$DAYOFBSTAGE <- as.numeric(as.character(dframe2$DAYOFBSTAGE))

dframe2$DAYOFSHIFT <- as.numeric(as.character(dframe2$DAYOFSHIFT))

dframe2$LENGTHOFSHIFT <- as.numeric(as.character(dframe2$LENGTHOFSHIFT))

# CREATE NEW COLUMN FOR THE PROPORTION OF INCUBATIONS SHIFT THE ADULT HAS COMPLETED UPON WEIGHING

dframe2$XSHIFT<-(dframe2$DAYOFSHIFT/dframe2$LENGTHOFSHIFT)*100

dframe2<-subset(dframe2, XSHIFT!="NA")#remove na values

#n=387

##~~~~~~~~~~~~~~~~~~~~~~~~~~~~~~~~~~~~~~~~~~~~~~~~~~~~~~~~~~~~~~~~~~~~~~~~~~~~~~

## SIMPLE SUMMARIES ----

##~~~~~~~~~~~~~~~~~~~~~~~~~~~~~~~~~~~~~~~~~~~~~~~~~~~~~~~~~~~~~~~~~~~~~~~~~~~~~~

#INCUBATION SHIFT SUMMARY TABLE

output4<- dframe2 %>% mutate(count=1) %>% group_by(LENGTHOFSHIFT) %>%

summarise(n=sum(count), meanw=mean(Weight),Weightsd=sd(Weight),wmin=min(Weight), wmax=max(Weight))

output4

#differences in mass by sex

output4a<- dframe2 %>% mutate(count=1) %>% group_by(Sex) %>%

summarise(n=sum(count), meanw=mean(Weight),Weightsd=sd(Weight),wmin=min(Weight), wmax=max(Weight))

output4a

#differences in adult mass by hatching outcome

output4b<- dframe2 %>% mutate(count=1) %>% group_by(HATCHED) %>%

summarise(n=sum(count), meanw=mean(Weight),Weightsd=sd(Weight),wmin=min(Weight), wmax=max(Weight))

output4b

#SUBSET THE INCUBATIONS SHIFTS INTO EITHER SHORT OR LONG

dframe2a<-subset(dframe2,LENGTHOFSHIFT <7 )

dframe2b<-subset(dframe2,LENGTHOFSHIFT >6 )

#SUMMARY OF SHORT INC SHIFTS

output5<-dframe2b %>% mutate(count=1) %>% summarise(n=sum(count), meanweight=mean(Weight),sd=sd(Weight))

output5 #n=248 mean= 725.9476 sd= 77.11006

#SUMMARIY OF LONG SHIFTS

output6<-dframe2a %>% mutate(count=1) %>% summarise(n=sum(count), meanweight=mean(Weight),sd=sd(Weight))

output6 #n=139 mean=740.7194 sd= 63.13535

#SUBSET THE PROPORTION OF INCUBATION SHIFT COMPLETED INTO THE FIRST HALF AND LAST HALF

dframe2c<-subset(dframe2,XSHIFT <50 )

dframe2d<-subset(dframe2,XSHIFT >50 )

#SUMMARY OF THE FIRST HALF OF INCUBATION SHIFTS

output7<-dframe2c %>% mutate(count=1) %>% summarise(n=sum(count), meanweight=mean(Weight),sd=sd(Weight))

output7# FIRST HALF OF INCUBATION SHIFT n=248 mean =762.9032 sd=57.26244

output8<-dframe2d %>% mutate(count=1) %>% summarise(n=sum(count), meanweight=mean(Weight),sd=sd(Weight))

output8 #LAST HALF OF INCUBATION SHIFT, n=117 mean =663.6325 SD= 55.99655

##~~~~~~~~~~~~~~~~~~~~~~~~~~~~~~~~~~~~~~~~~~~~~~~~~~~~~~~~~~~~~~~~~~~~~~~~~~~~~~

## STATISTICS: WHAT FACTORS INCLUENCE MASS DURING INCUBATION ----

##~~~~~~~~~~~~~~~~~~~~~~~~~~~~~~~~~~~~~~~~~~~~~~~~~~~~~~~~~~~~~~~~~~~~~~~~~~~~~~

m0 <- glmer(Weight ~ 1+ (1|Ring_Nr)+ (1|NEST_ID),family=Gamma(link="log"), data=dframe2)

m1 <- glmer(Weight ~ Fyear+ (1|Ring_Nr)+ (1|NEST_ID),family=Gamma(link="log"), data=dframe2)

m2<- glmer(Weight ~ scale(XSHIFT)+(1|Ring_Nr)+ (1|NEST_ID),family=Gamma(link="log"), data=dframe2)

m3 <- glmer(Weight ~ scale(DAYOFBSTAGE)+ (1|Ring_Nr)+ (1|NEST_ID),family=Gamma(link="log"), data=dframe2)

m4 <- glmer(Weight ~ scale(LENGTHOFSHIFT) + (1|Ring_Nr)+ (1|NEST_ID),family=Gamma(link="log"), data=dframe2)

lrtest(m0, m1) # YEAR #CHISQ= 3.1621 p= 0.5311 df= 4,n=387 no dif in body con during inc between years

lrtest(m0, m2) # XSHIFT ## CHISQ= 280.16 p=< 2.2e-16 *** DF=1, N=387 # sig neg affect of porportion of shift completed and weight during incubation

lrtest(m0, m3) # DOFIP ## CHISQ=8.3425 p= 0.003873 ** n=387, df=1 # neg relationship the further into incubaion period the lower the body con

lrtest(m0, m4) # LOFSHFT ## CHISQ=4.481 P=0.03427 * N=387,df=1 neg relationship, the longer the shift the lighter the adult

dframe2a<-subset(dframe2,Sex!="")#n=352 # REMOVE ADULTS OF UNKNOWN SEX

m6a <- glmer(Weight ~ 1+ (1|Ring_Nr)+ (1|NEST_ID),family=Gamma(link="log"), data=dframe2a)

m6b <- glmer(Weight ~ Sex+(1|Ring_Nr)+ (1|NEST_ID),family=Gamma(link="log"), data=dframe2a)

lrtest(m6a, m6b) # SEX # df=1, CHISQ= 0.058 p= 0.8097 ,n=352

#no significant diffance in body condition between sexes during incubation

dframe2a <- subset(dframe2, dframe2$HATCHED !="UNK")# n=378 # REMOVE ADULTS WHERE THE INCUBATION OUTCOME WAS UNKNOWN

m7a <- glmer(Weight ~ 1+ (1|Ring_Nr)+ (1|NEST_ID),family=Gamma(link="log"), data=dframe2a)

m7b <- glmer(Weight ~ HATCHED+(1|Ring_Nr)+ (1|NEST_ID),family=Gamma(link="log"), data=dframe2a)

lrtest(m7a, m7b) # HATCHED #CHISQ=0.5745, P= 0.4485 df1 n=378 , DF=1

# NO SIGNIFICANT DIFFERENCE IN BODY CONDITION BETWEEN NESTS THAT HATCHED AND THOSE THAT FAILED

##~~~~~~~~~~~~~~~~~~~~~~~~~~~~~~~~~~~~~~~~~~~~~~~~~~~~~~~~~~~~~~~~~~~~~~~~~~~~~~

## STATISTICS: GLMM MODEL SELECTION FOR ADULT MASS DURING INCUBATION ----

##~~~~~~~~~~~~~~~~~~~~~~~~~~~~~~~~~~~~~~~~~~~~~~~~~~~~~~~~~~~~~~~~~~~~~~~~~~~~~~

#wHICH VARIABLES ARE ASSOCIATED WITH RED-BILLED TROPICBIRD ADULT MASS DURING INCUBATION?

GLOBAL <- glmer(Weight ~ scale(XSHIFT)+scale(LENGTHOFSHIFT)+ scale(DAYOFBSTAGE)

+scale(LENGTHOFSHIFT):scale(XSHIFT)+scale(XSHIFT):scale(DAYOFBSTAGE)+scale(LENGTHOFSHIFT):scale(DAYOFBSTAGE)

+ (1|Ring_Nr)+ (1|NEST_ID), family=Gamma(link="log"), data=dframe2)

INC1 <<- glmer(Weight ~ scale(XSHIFT)+scale(LENGTHOFSHIFT)+ scale(DAYOFBSTAGE)

+scale(LENGTHOFSHIFT):scale(XSHIFT)+scale(LENGTHOFSHIFT):scale(DAYOFBSTAGE)

+ (1|Ring_Nr)+ (1|NEST_ID), family=Gamma(link="log"), data=dframe2)

INC2 <<- glmer(Weight ~ scale(XSHIFT)+scale(LENGTHOFSHIFT)

+scale(LENGTHOFSHIFT):scale(XSHIFT)+scale(LENGTHOFSHIFT):scale(DAYOFBSTAGE)

+ (1|Ring_Nr)+ (1|NEST_ID), family=Gamma(link="log"), data=dframe2)

INC3 <<- glmer(Weight ~ scale(XSHIFT)+scale(LENGTHOFSHIFT)

+scale(LENGTHOFSHIFT):scale(XSHIFT)

+ (1|Ring_Nr)+ (1|NEST_ID), family=Gamma(link="log"), data=dframe2)

r.squaredGLMM(INC3)#delta R2M= 0.4669612 R2C=0.7097326

#.................TABLE 1 MODEL SELECTION OUTPUT.................####

AIC_fTABLE<-aictab(cand.set=list(GLOBAL,INC1,INC2,INC3),modnames=c('GLOBAL','XSHIFT:BSTAGE','BSTAGE', 'LENGTHOFSHIFT:BSTAGE'),sort = TRUE, c.hat = 1, second.ord = TRUE, nobs = NULL)

AIC_fTABLE

summary(INC3)

##~~~~~~~~~~~~~~~~~~~~~~~~~~~~~~~~~~~~~~~~~~~~~~~~~~~~~~~~~~~~~~~~~~~~~~~~~~~~~~

## FIGURE A1 INTERACTION PLOT FOR MODEL3 IN TABLE 1 ----

##~~~~~~~~~~~~~~~~~~~~~~~~~~~~~~~~~~~~~~~~~~~~~~~~~~~~~~~~~~~~~~~~~~~~~~~~~~~~~~

#RUN MODEL 3 AS A GAMM

INC<-gamm4(Weight ~s(XSHIFT, k=6, bs="tp")+s(LENGTHOFSHIFT, k=6, bs="tp")+LENGTHOFSHIFT:XSHIFT, random = ~ (1|NEST_ID)+(1|Ring_Nr), na.action=na.exclude, family=Gamma(link="log"),data=dframe2)

summary(INC$gam)

gam.check(INC$gam)

plot(INC$gam)

vis.gam(INC$gam)

###~~~~~~~~~~~~~~~

## ~ FIGA1A ----

##~~~~~~~~~~~~~~~

# FIGURE A1A PROPORTION OF SHIFT EFFECT ##

#................make a dataframe of predictions.................

pdat <- expand.grid(XSHIFT= seq(8,100, 2),LENGTHOFSHIFT= 1) ; pdat

pred <- predict (INC$gam, newdata = pdat, na.rm = T, type= "response", se.fit = TRUE) ; pred

predframemin <- data.frame (pdat, preds = pred); predframemin

predframemin$uppersemin <- (predframemin$preds.fit + predframemin$preds.se.fit)

predframemin$lowersemin <- (predframemin$preds.fit - predframemin$preds.se.fit)

pdat <- expand.grid(XSHIFT= seq(8,100, 2),LENGTHOFSHIFT= 13) ; pdat

pred <- predict (INC$gam, newdata = pdat, na.rm = T, type= "response", se.fit = TRUE) ; pred

predframemax <- data.frame (pdat, preds = pred); predframemax

predframemax$uppersemax <- (predframemax$preds.fit + predframemax$preds.se.fit)

predframemax$lowersemax <- (predframemax$preds.fit - predframemax$preds.se.fit)

#........................plot predictions........................

A<-ggplot() +

geom_point(data=dframe2, aes(y=Weight, x=XSHIFT), col="black", fill=NA, pch=1,size=2)+

geom_ribbon(aes(ymin = lowersemin, ymax = uppersemin,x = XSHIFT),data=predframemin, alpha = 0.5, fill="gold" )+

geom_line(data=predframemin, mapping=aes(y=preds.fit, x=XSHIFT),col="gold2", size=1.5 , alpha = 0.75)+

geom_ribbon(aes(ymin = lowersemax, ymax = uppersemax,x = XSHIFT),data=predframemax, alpha = 0.5, fill="royalblue" )+

geom_line(data=predframemax, mapping=aes(y=preds.fit, x=XSHIFT),col="royalblue", size=1.5 , alpha = 0.75)+

scale_y_continuous(name="Adult mass (g)", breaks =seq(500,1000,100),expand=c(0,0), limits=c(500,1000))+

scale_x_continuous(breaks=seq(0,100,10),limits=c(0,102), expand=c(0,0), name="Proportion of completed incubation shift (%)")+

ggtitle("a).") +

theme_ipsum(grid=FALSE, axis=TRUE,

axis_title_size = 14,

base_size = 12,

axis_title_face = "bold",

axis_title_just = "mc",

axis_col = "black",

ticks=TRUE)+

theme (plot.title = element_text(size=14, face="bold", colour="black", vjust = 2, hjust=-0.08),

plot.margin = unit(c(.5, .5, .5, .5), "cm"))

A

###~~~~~~~~~~~~~~~

## ~ FIGA1B ----

##~~~~~~~~~~~~~~~

#LENGTH OF INCUBATINS SHIFT AFFECT

#................make a dataframe of predictions.................

pdat <- expand.grid(LENGTHOFSHIFT= seq(1,13, 1),XSHIFT= 8.333) ; pdat

pred <- predict (INC$gam, newdata = pdat, na.rm = T, type= "response", se.fit = TRUE) ; pred

predframemin <- data.frame (pdat, preds = pred); predframemin

predframemin$upperse <- (predframemin$preds.fit + predframemin$preds.se.fit)

predframemin$lowerse <- (predframemin$preds.fit - predframemin$preds.se.fit)

pdat <- expand.grid(LENGTHOFSHIFT= seq(1,13, 1),XSHIFT= 100) ; pdat

pred <- predict (INC$gam, newdata = pdat, na.rm = T, type= "response", se.fit = TRUE) ; pred

predframemax <- data.frame (pdat, preds = pred); predframemax

predframemax$upperse <- (predframemax$preds.fit + predframemax$preds.se.fit)

predframemax$lowerse <- (predframemax$preds.fit - predframemax$preds.se.fit)

#........................plot predictions........................

B<-ggplot() +

geom_point(data=dframe2, aes(y=Weight, x=LENGTHOFSHIFT), col="black", fill=NA, pch=1,size=3)+

geom_ribbon(aes(ymin = lowerse, ymax = upperse,x = LENGTHOFSHIFT),data=predframemin, alpha = 0.5, fill="gold" )+

geom_line(data=predframemin, mapping=aes(y=preds.fit, x=LENGTHOFSHIFT),col="gold2", size=1.5 , alpha = 0.75)+

geom_ribbon(aes(ymin = lowerse, ymax = upperse,x = LENGTHOFSHIFT),data=predframemax, alpha = 0.5, fill="royalblue" )+

geom_line(data=predframemax, mapping=aes(y=preds.fit, x=LENGTHOFSHIFT),col="royalblue", size=1.5 , alpha = 0.75)+

scale_y_continuous(name="Adult mass (g)", breaks =seq(500,1000,100),expand=c(0,0), limits=c(500,1000))+

scale_x_continuous(breaks=seq(0,13,1),limits=c(0,13.75), expand=c(0,0), name="Length of incubation shift (days)")+

ggtitle("b).") +

theme_ipsum(grid=FALSE, axis=TRUE,

axis_title_size = 14,

base_size = 12,

axis_title_face = "bold",

axis_title_just = "mc",

axis_col = "black",

ticks=TRUE)+

theme (plot.title = element_text(size=14, face="bold", colour="black", vjust = 2, hjust= -0.08),

plot.margin = unit(c(.5, .5, .5, .5), "cm"))

B

#..................combine plots into one frame..................

FIGA1 <- ggarrange(ggarrange(A, B, labels = NULL, nrow=2))

FIGA1

##~~~~~~~~~~~~~~~~~~~~~~~~~~~~~~~~~~~~~~~~~~~~~~~~~~~~~~~~~~~~~~~~~~~~~~~~~~~~~~

## STATISTICS: INCUBATION SHIFTS ----

##~~~~~~~~~~~~~~~~~~~~~~~~~~~~~~~~~~~~~~~~~~~~~~~~~~~~~~~~~~~~~~~~~~~~~~~~~~~~~~

#............................LOAD DATA...........................####

#SET THE WORKING DIRECTORY

#setwd("C:\\Users\\X")

#READ THE CSV FILE

dframe2 <- read.csv("INCUBATION_ROUTINE.csv") # Read in the adult weights data n=323

#..........................FORMAT DATA ..........................####

dframe2$DAY.OF.REGIME<-as.character(dframe2$DAY.OF.REGIME)

dframe2$DAY.OF.REGIME<-as.numeric(dframe2$DAY.OF.REGIME)

dframe2$YEAR<-as.factor(dframe2$YEAR)

#create dataframe for inc shift sequence

dframe3<-subset(dframe2, SHIFT_NUMBER !="N/A")# REMOVE RECORDS WHERE THE SHIFT NUMBER COULD NOT BE IDENTIFED

dframe3$SHIFT_NUMBER<-as.character(dframe3$SHIFT_NUMBER)

dframe3$SHIFT_NUMBER<-as.numeric(dframe3$SHIFT_NUMBER)

summary(dframe3)#N=221

dframe2<- subset(dframe2, DAY.OF.REGIME<43)#REMOVE SHIFTS THAT START PAST DAY 43 OF INC PERIOD

#N= 314

#SUBSET THE DATA TO REMOVE ADULTS OF UNKNOWN SEX OR HATCHING OUTCOME, n=289

dframe2a<- subset(dframe2, SEX!="UNK")#REMOVE ADULTS OF UNKNOWN SEX

dframe2a<- subset(dframe2a, HATCHED!="UNK")#N=289 REMOVE RECORDS WHERE THE INCUBATION OUTCOME IS UNKNOWN

##~~~~~~~~~~~~~~~~~~~~~~~~~~~~~~~~~~~~~~~~~~~~~~~~~~~~~~~~~~~~~~~~~~~~~~~~~~~~~~

## SIMPLE SUMMARIES ----

##~~~~~~~~~~~~~~~~~~~~~~~~~~~~~~~~~~~~~~~~~~~~~~~~~~~~~~~~~~~~~~~~~~~~~~~~~~~~~~

#................TABLE 2 INCUBATION SHIFT DURATION...............####

#SHIFT SEQUENCE SUMMARY # TABLE 2 ###

output9<- dframe3 %>% mutate(count=1) %>% group_by(SHIFT_NUMBER,SEX) %>%

summarise(n=sum(count), meanshiftlength=mean(SHIFT),shiftsd=sd(SHIFT),shiftmin=min(SHIFT), shiftmax=max(SHIFT))

output9 # n=221

#HOW MANY INDIVIDUAL NESTS WERE MONITORED

output10<- dframe3 %>% mutate(count=1) %>% group_by(NEST_ID) %>% summarise(n=sum(count))

output10

summary(output10)# n= 37 nests

#DIFFERENCES IN INCUBATION SHIFT LENGTH BETWEEN SEXES

output11<- dframe2a %>% mutate(count=1) %>% group_by(SEX) %>%

summarise(n=sum(count), mean=mean(SHIFT),sd=sd(SHIFT),min=min(SHIFT), max=max(SHIFT))

output11 #n=289, F=143, M=146

#DIFFERENCES BETWEEN YEARS

output12<- dframe2a %>% mutate(count=1) %>% group_by(YEAR) %>%

summarise(n=sum(count), mean=mean(SHIFT),sd=sd(SHIFT),min=min(SHIFT), max=max(SHIFT))

output12 #n=289

#DIFFERENCES BETWEEN SUCCESSFULLY HATCHED NESTS AND FAILED NESTS

output13<- dframe2a %>% mutate(count=1) %>% group_by(HATCHED) %>%

summarise(n=sum(count), mean=mean(SHIFT),sd=sd(SHIFT),min=min(SHIFT), max=max(SHIFT))

output13#n=289

#AVERAGE SHIFT LENGTH DURING INCUBATION

output14<- dframe2 %>% mutate(count=1) %>%

summarise(n=sum(count), mean=mean(SHIFT),sd=sd(SHIFT),min=min(SHIFT), max=max(SHIFT))

output14# MEAN =6.045446 SD=2.757187 MIN=0.01MAX= 11.89

##~~~~~~~~~~~~~~~~~~~~~~~~~~~~~~~~~~~~~~~~~~~~~~~~~~~~~~~~~~~~~~~~~~~~~~~~~~~~~~

## STATISTICS: GAMM MODEL SELECTION PROCESS FOR INCUBATION SHIFTS ----

##~~~~~~~~~~~~~~~~~~~~~~~~~~~~~~~~~~~~~~~~~~~~~~~~~~~~~~~~~~~~~~~~~~~~~~~~~~~~~~

# WHICH VARIABLES WERE ASSOCIATE WITH INCUBATION SHIFT LENGTH IN BREEDING RED-BILLED TROPICBIRDS?

# STEP-WISE DELETION MODEL SELECTION PROCESS

model1 <- gamm4(SHIFT ~ s(DAY.OF.REGIME,k=6, fx=FALSE, by=SEX) +

+s(DAY.OF.REGIME,k=6, fx=FALSE) + HATCHED+ SEX+ YEAR,

random= ~(1|ADULT) + (1|NEST_ID), data=dframe2a,family=Gamma(link="log"), REML=TRUE) #

summary(model1$gam)

summary(model1$mer)#AIC 1453.2

anova(model1$gam)

model2 <- gamm4(SHIFT ~ s(DAY.OF.REGIME,k=6, fx=FALSE, by=SEX) + HATCHED+ SEX+ YEAR,

# +s(DAY.OF.REGIME,k=6, fx=FALSE)+

random= ~(1|ADULT) + (1|NEST_ID), data=dframe2a,family=Gamma(link="log"), REML=TRUE) #

summary(model2$gam)

summary(model2$mer)# AIC 1451.2

anova(model2$gam)

#FINAL MODEL ##

model3 <- gamm4(SHIFT ~ s(DAY.OF.REGIME,k=6, fx=FALSE, by=SEX) + SEX+ YEAR,

random= ~(1|ADULT) + (1|NEST_ID), data=dframe2a,family=Gamma(link="log"), REML=TRUE) #

summary(model3$gam)

anova(model3$gam)

summary(model3$mer)# AIC 1450.3

# MODEL NOT INCLUDED AS DID NOT IMPROVE MODEL PERFORMANCE

#model4 <- gamm4(SHIFT ~ s(DAY.OF.REGIME,k=6, fx=FALSE, by=SEX) + SEX,#+ YEAR,

# random= ~(1|ADULT) + (1|NEST_ID), data=dframe2a,family=Gamma(link="log"), REML=TRUE)

#summary(model4$gam)

#anova(model4$gam)

#summary(model4$mer)# AIC 1450.6 HIGHER SO KEEP YEAR IN FINAL MODEL

##~~~~~~~~~~~~~~~~~~~~~~~~~~~~~~~~~~~~~~~~~~~~~~~~~~~~~~~~~~~~~~~~~~~~~~~~~~~~~~

## FIGURE 4 INTERACTION PLOT BETWEEN SEX, INCUBATION PERIOS AND SHIFT LENGTH----

##~~~~~~~~~~~~~~~~~~~~~~~~~~~~~~~~~~~~~~~~~~~~~~~~~~~~~~~~~~~~~~~~~~~~~~~~~~~~~~

pdat <- expand.grid(DAY.OF.REGIME = seq(0,43, 1), SEX="F", YEAR="2013"); pdat

pred <- predict (model3$gam, newdata = pdat, na.rm = T,type= "response", se.fit = TRUE) ; pred

predframef <- data.frame (pdat, preds = pred); predframef

predframef$upperse <- (predframef$preds.fit + predframef$preds.se.fit)

predframef$lowerse <- (predframef$preds.fit - predframef$preds.se.fit)

pdat <- expand.grid(DAY.OF.REGIME = seq(0,43, 1), SEX="M", YEAR="2013" ); pdat

pred <- predict (model3$gam, newdata = pdat, na.rm = T,type= "response", se.fit = TRUE) ; pred

predframem <- data.frame (pdat, preds = pred); predframem

predframem$upperse <- (predframem$preds.fit + predframem$preds.se.fit)

predframem$lowerse <- (predframem$preds.fit - predframem$preds.se.fit)

colours <- c("F" = "red","M" = "blue")

ggplot() +

geom_point(data=dframe2a, aes(y=SHIFT, x=DAY.OF.REGIME, col=SEX), fill=NA,size=3, alpha=0.5)+

geom_ribbon(aes(ymin = lowerse, ymax = upperse,x = DAY.OF.REGIME),data=predframef, alpha = 0.5, fill="red" )+

geom_ribbon(aes(ymin = lowerse, ymax = upperse,x = DAY.OF.REGIME),data=predframem, alpha = 0.5, fill="blue" )+

geom_line(data=predframef, mapping=aes(y=preds.fit, x=DAY.OF.REGIME),col="red", size=1.5 )+

geom_line(data=predframem, mapping=aes(y=preds.fit, x=DAY.OF.REGIME),col="blue", size=1.5 )+

scale_y_continuous(name="Incubation shift length (days)", breaks =seq(0,13,1),expand=c(0,0), limits=c(-0.25,13))+

scale_x_continuous(breaks=seq(0,43,10),limits=c(-0.5,43.5), expand=c(0,0), name="Incubation period (days)")+

scale_color_manual(values=colours, labels = c("Female", "Male"), name="", guide="legend")+

guides(colour=guide_legend(override.aes =list(size = c(6,6),linetype=c(1,1),shape=c(15,15),alpha=c(1,1))))+

theme_ipsum(grid=FALSE, axis=TRUE,

axis_title_size = 14,

base_size = 12,

axis_title_face = "bold",

axis_title_just = "mc",

axis_col = "black",

ticks=TRUE)+

theme(legend.position=c(0.85,0.95),legend.text=element_text(size=12, colour="black"))

##~~~~~~~~~~~~~~~~~~~~~~~~~~~~~~~~~~~~~~~~~~~~~~~~~~~~~~~~~~~~~~~~~~~~~~~~~~~~~~

## STATISTICS: MASS LOSS DURING INCUBATION ----

##~~~~~~~~~~~~~~~~~~~~~~~~~~~~~~~~~~~~~~~~~~~~~~~~~~~~~~~~~~~~~~~~~~~~~~~~~~~~~~

#............................LOAD DATA...........................####

#SET THE WORKING DIRECTORY

#setwd("C:\\Users\\x")

#READ THE CSV FILE

dframe3 <- read.csv("ADULT_MASS_LOSS_DURING_INCUBATION.csv")

#..........................FORMAT DATA ..........................####

# Extract date values

date <- parse_date_time(dframe3$Date, "dmy")

date

# Year as a number

dframe3$year <- year(date)

dframe3$year

# Year as a factor

dframe3$Fyear <- as.factor(dframe3$year)

summary(dframe3$Fyear) # request sample sizes

dframe3$DAYOFBSTAGE <- as.numeric(dframe3$DAYOFBSTAGE)

#N=88

names(dframe3)

##~~~~~~~~~~~~~~~~~~~~~~~~~~~~~~~~~~~~~~~~~~~~~~~~~~~~~~~~~~~~~~~~~~~~~~~~~~~~~~

## SIMPLE SUMMARIES ----

##~~~~~~~~~~~~~~~~~~~~~~~~~~~~~~~~~~~~~~~~~~~~~~~~~~~~~~~~~~~~~~~~~~~~~~~~~~~~~~

# AVERAGE PROPORTION OF MASS LOSS PER DAY DURING INCCUBATION SHIFTS

output15<- dframe3 %>% mutate(count=1) %>% #group_by(HATCHED) %>%

summarise(n=sum(count), mean=mean(X.BODYMASSLOSSPERDAY),Weightsd=sd(X.BODYMASSLOSSPERDAY),wmin=min(X.BODYMASSLOSSPERDAY), wmax=max(X.BODYMASSLOSSPERDAY))

output15

#AVERAGE WEIGHT OF ADULTS PER YEAR DURING INCUBATION

output16<-dframe3 %>% mutate(count=1) %>% group_by(Fyear) %>% summarise(n=sum(count), meanweight=mean(Weight),max_distsd=sd(Weight))

output16

#SUBSET THE DATA INTO TWO PORTIONS ON INCUBATION SHIFT COMPLETED

dframe3b<-subset(dframe3,X.SHIFT > 50 )#n=78 last half of shift

dframe3c<-subset(dframe3,X.SHIFT < 50 )#n=6 first half of shift

#MASS LOSS PER DAY IN THE LAST HALF OF INCUBATION SHIFT

output17a<- dframe3b %>% mutate(count=1) %>% #group_by(X.SHIFT) %>%

summarise(n=sum(count), meanw=mean(MASSLOSSPERDAYg),Weightsd=sd(MASSLOSSPERDAYg),wmin=min(MASSLOSSPERDAYg), wmax=max(MASSLOSSPERDAYg))

output17a

#MASS LOSS PER DAY IN THE FIRST HALF OF INCUBATION SHIFT

output17b<- dframe3c %>% mutate(count=1) %>%# group_by(X.SHIFT) %>%

summarise(n=sum(count), meanw=mean(MASSLOSSPERDAYg),Weightsd=sd(MASSLOSSPERDAYg),wmin=min(MASSLOSSPERDAYg), wmax=max(MASSLOSSPERDAYg))

output17b

##~~~~~~~~~~~~~~~~~~~~~~~~~~~~~~~~~~~~~~~~~~~~~~~~~~~~~~~~~~~~~~~~~~~~~~~~~~~~~~

## STATISTICS: GLMMS MODELING MASS LOSS PER DAY DURING INCUBATION ----

##~~~~~~~~~~~~~~~~~~~~~~~~~~~~~~~~~~~~~~~~~~~~~~~~~~~~~~~~~~~~~~~~~~~~~~~~~~~~~~

#WHICH VARAIBLES ARE ASSOCIATED WITH MASS LOSS PER DAY DURING INCUBATION?

ML0 <- glmer(X.BODYMASSLOSSPERDAY ~1 + (1|NEST_ID) + (1|Ring_Nr), family=Gamma(link="log"), data=dframe3)

ML1 <- glmer(X.BODYMASSLOSSPERDAY ~Sex + (1|NEST_ID) + (1|Ring_Nr), family=Gamma(link="log"), data=dframe3)

ML2 <- glmer(X.BODYMASSLOSSPERDAY ~Fyear + (1|NEST_ID) + (1|Ring_Nr), family=Gamma(link="log"), data=dframe3)

ML3 <- glmer(X.BODYMASSLOSSPERDAY ~HATCHED + (1|NEST_ID) + (1|Ring_Nr), family=Gamma(link="log"), data=dframe3)

ML4 <- glmer(X.BODYMASSLOSSPERDAY ~scale(X.SHIFT) + (1|NEST_ID) + (1|Ring_Nr), family=Gamma(link="log"), data=dframe3)

ML5<- glmer(X.BODYMASSLOSSPERDAY ~scale(LENGTHOFSHIFT) + (1|NEST_ID) + (1|Ring_Nr), family=Gamma(link="log"), data=dframe3)

dframe3a<-subset(dframe3,DAYOFBSTAGE!="NA" )# n=80

ML0a <- glmer(X.BODYMASSLOSSPERDAY ~1 + (1|NEST_ID) + (1|Ring_Nr), family=Gamma(link="log"), data=dframe3a)

ML6 <- glmer(X.BODYMASSLOSSPERDAY ~scale(DAYOFBSTAGE) + (1|NEST_ID) + (1|Ring_Nr), family=Gamma(link="log"), data=dframe3a)

lrtest(ML0, ML1)# SEX #DF=1 X2=0.1892 p=0.6636, n=88

lrtest(ML0, ML2)# YEAR #DF=4 X2=1.3304 p=0.8562, n=88

lrtest(ML0, ML3)# HATCHING OUTCOME #DF=1 X2=0.0802 p=0.777, n=88

lrtest(ML0, ML4)# PROPORTION OF SHIFT COMPLETED # DF=1 X2=9.6895 P= 0.001853 ** # ONLY ONE SIGNIFICANT##

lrtest(ML0, ML5)# LENGTH OF SHIFT #DF =1 X2=0.6033 P= 0.4373

lrtest(ML0a, ML6)# DAY OF INCUBATION PERIOD #DF=1 X2=0.199 0.6555 n=81

summary(ML4)

##~~~~~~~~~~~~~~~~~~~~~~~~~~~~~~~~~~~~~~~~~~~~~~~~~~~~~~~~~~~~~~~~~~~~~~~~~~~~~~

## STATISTICS: MODEL SELECTION FOR MASS LOSS PER DAY DURING INCUBATION ----

##~~~~~~~~~~~~~~~~~~~~~~~~~~~~~~~~~~~~~~~~~~~~~~~~~~~~~~~~~~~~~~~~~~~~~~~~~~~~~~

dframe3<-subset(dframe3,DAYOFBSTAGE!="NA" ) # n=81

GLOBAL <- glmer(X.BODYMASSLOSSPERDAY ~ scale(X.SHIFT)+scale(LENGTHOFSHIFT)+ scale(DAYOFBSTAGE)

+scale(LENGTHOFSHIFT):scale(X.SHIFT)+scale(X.SHIFT):scale(DAYOFBSTAGE)+scale(LENGTHOFSHIFT):scale(DAYOFBSTAGE)

+ (1|Ring_Nr)+ (1|NEST_ID), family=Gamma(link="log"), data=dframe3)

INCML1 <- glmer(X.BODYMASSLOSSPERDAY ~ scale(X.SHIFT)+scale(LENGTHOFSHIFT)+ scale(DAYOFBSTAGE)

+scale(LENGTHOFSHIFT):scale(X.SHIFT)+scale(X.SHIFT):scale(DAYOFBSTAGE)+

+ (1|Ring_Nr)+ (1|NEST_ID), family=Gamma(link="log"), data=dframe3)

INCML2 <- glmer(X.BODYMASSLOSSPERDAY ~ scale(X.SHIFT)+scale(LENGTHOFSHIFT)+ scale(DAYOFBSTAGE)

+scale(X.SHIFT):scale(DAYOFBSTAGE)+ (1|Ring_Nr)+ (1|NEST_ID), family=Gamma(link="log"), data=dframe3)

INCML3 <- glmer(X.BODYMASSLOSSPERDAY ~ scale(X.SHIFT)+scale(LENGTHOFSHIFT)+ scale(DAYOFBSTAGE)

+ (1|Ring_Nr)+ (1|NEST_ID), family=Gamma(link="log"), data=dframe3)

INCML4 <- glmer(X.BODYMASSLOSSPERDAY ~ scale(X.SHIFT)+scale(LENGTHOFSHIFT)

+ (1|Ring_Nr)+ (1|NEST_ID), family=Gamma(link="log"), data=dframe3)

#INCML5 <- glmer(X.BODYMASSLOSSPERDAY ~ scale(X.SHIFT)

# + (1|Ring_Nr)+ (1|NEST_ID), family=Gamma(link="log"), data=dframe3)

summary(INCML4)

r.squaredGLMM(INCML4)#delta r2m= 0.1904281, r2c=0.2018577

#.................TABLE 4 MODEL SELECTION OUTPUT.................####

AIC_INCMASSLOSSTABLE<-aictab(cand.set=list(GLOBAL,INCML1,INCML2,INCML3,INCML4),modnames=c('GLOBAL','LOFSHIFTXBSTAGE','XSTAGEXLOFSHIFT','XSHIFT:BSTAGE','BSTAGE'),sort = TRUE, c.hat = 1, second.ord = TRUE, nobs = NULL)

AIC_INCMASSLOSSTABLE

##~~~~~~~~~~~~~~~~~~~~~~~~~~~~~~~~~~~~~~~~~~~~~~~~~~~~~~~~~~~~~~~~~~~~~~~~~~~~~~

## ADULTS REARING CHICKS ----

##~~~~~~~~~~~~~~~~~~~~~~~~~~~~~~~~~~~~~~~~~~~~~~~~~~~~~~~~~~~~~~~~~~~~~~~~~~~~~~

#............................LOAD DATA...........................####

#SET THE WORKING DIRECTORY

#setwd("C:\\Users\\x")

#READ THE CSV FILE

dframe4 <- read.csv("CHICK_REARING_ADULT_ATTENDANCE.csv")

dframe5 <- read.csv("ADULT_MASS_DURING_CHICK_REARING.csv")

#..........................FORMAT DATA ..........................####

#arrange dates

date <- parse_date_time(dframe5$Date, "dmy")

# Year as a number

dframe5$year <- year(date)

# Year as a factor

dframe5$Fyear <- as.factor(dframe5$year)

dframe5$DAYOFBSTAGE <- as.numeric(as.character(dframe5$DAYOFBSTAGE))

dframe5$Fyear <- relevel(dframe5$Fyear, ref="2015")# relevels model so ref year is 2015

dframe5$WEEKOFCRF<-as.factor(dframe5$WEEKOFCR)

dframe5$WEEKOFCR<-as.numeric(dframe5$WEEKOFCR)

dframe5$FIVEDAYINTF<-as.factor(dframe5$FIVEDAYINT)

#n=290

##~~~~~~~~~~~~~~~~~~~~~~~~~~~~~~~~~~~~~~~~~~~~~~~~~~~~~~~~~~~~~~~~~~~~~~~~~~~~~~

## SIMPLE SUMMARIES ----

##~~~~~~~~~~~~~~~~~~~~~~~~~~~~~~~~~~~~~~~~~~~~~~~~~~~~~~~~~~~~~~~~~~~~~~~~~~~~~~

#AVERAGE MASS OF ADULTS AT FIVE DAY INTERVALS DURING THE CHICK REARING PERIOD

CRmass<-dframe5 %>% mutate(count=1) %>% group_by(FIVEDAYINT) %>% summarise(n=sum(count), meanweight=mean(Weight),sd=sd(Weight))

CRmass

#AVERAGE MASS OF ADULTS DURING EACH WEEK OF THE CHICK REARING PERIOD

CRmass<-dframe5 %>% mutate(count=1) %>% group_by(WEEKOFCR) %>% summarise(n=sum(count), meanweight=mean(Weight),sd=sd(Weight))

CRmass

##~~~~~~~~~~~~~~~~~~~~~~~~~~~~~~~~~~~~~~~~~~~~~~~~~~~~~~~~~~~~~~~~~~~~~~~~~~~~~~

## STATISTICS: DIFFERNECES IN ADULT MASS AT STH AND ASC ----

##~~~~~~~~~~~~~~~~~~~~~~~~~~~~~~~~~~~~~~~~~~~~~~~~~~~~~~~~~~~~~~~~~~~~~~~~~~~~~~

#test for differences between two proportion of visits adults were present or not

#between the two islands, St Helena (STH) and Ascension island (ASC)

subset(dframe4, AGEGROUP< 11)

ASCP<-253

STHP<-738

ASCV<-708

STHV<-1343

prop.test(c(ASCP,STHP),c(ASCV, STHV))

#X-squared = 67.793, df = 1, p-value < 2.2e-16 ASC=0.3573446, STH= 0.5495160

#usin only the first five weeks as nests did not last longer than that

#test for differences in the proportion of visits parents were present or not and nesing outcome

FP<-316

FV<-451

XP<-334

XV<-454

prop.test(c(FP,XP),c(FV, XV))

#X-squared = 1.2033, df = 1, p-value = 0.2727, FLEDGED=0.7006652, FAILED= 0.7356828

##~~~~~~~~~~~~~~~~~~~~~~~~~~~~~~~~~~~~~~~~~~~~~~~~~~~~~~~~~~~~~~~~~~~~~~~~~~~~~~

## STATISTICS: BODY CONDITION DURING CHICK REARING ----

##~~~~~~~~~~~~~~~~~~~~~~~~~~~~~~~~~~~~~~~~~~~~~~~~~~~~~~~~~~~~~~~~~~~~~~~~~~~~~~

#DOES BODY CONDITION DIFFER BETWEEN SEXES, YEARS, WEEK OF CHICK AGE (FACTOR) OR DAY OF CHICK REARING PERIOD

dframe5$Fyear <- factor(dframe5$Fyear, levels=c("2013", "2014","2015","2016","2017"))

cr0 <- glmer(Weight~ 1 +

(1|Ring_Nr)+(1|NEST_ID),family=Gamma(link="log"), data=dframe5, na.action=na.exclude)

cr1 <- glmer(Weight~ scale(DAYOFBSTAGE) +

(1|Ring_Nr)+(1|NEST_ID),family=Gamma(link="log"), data=dframe5, na.action=na.exclude)

cr2 <- glmer(Weight~ Sex +

(1|Ring_Nr)+(1|NEST_ID),family=Gamma(link="log"), data=dframe5, na.action=na.exclude)

cr3 <- glmer(Weight~ Fyear+

(1|Ring_Nr)+(1|NEST_ID),family=Gamma(link="log"), data=dframe5, na.action=na.exclude)

cr4 <- glmer(Weight~ FLEDGED+

(1|Ring_Nr)+(1|NEST_ID),family=Gamma(link="log"), data=dframe5, na.action=na.exclude)

cr5 <- glmer(Weight~ WEEKOFCRF+

(1|Ring_Nr)+(1|NEST_ID),family=Gamma(link="log"), data=dframe5, na.action=na.exclude)

lrtest(cr0, cr1) #DAY OF THE CHICK REARING PERIOD, CHISQ= 0.0227, P= 0.8801, df=1 , N=290

lrtest(cr0, cr2) #BETWEEN SEXES, CHISQ=0.1572 P=0.6918, df=1, N=290

lrtest(cr0, cr3) #BETWEEN YEARS, CHISQ=1.6057, P= 0.8078, df=4, N=290

lrtest(cr0, cr4) #BETWEEN CHICKS THAT FLEDGED AND THOSE THAT FAILED, CHISQ=0.9113 P= 0.3398, DF=1, N=291

summary (cr5)

##~~~~~~~~~~~~~~~~~~~~~~~~~~~~~~~~~~~~~~~~~~~~~~~~~~~~~~~~~~~~~~~~~~~~~~~~~~~~~~

## FIGURE 5 PARENTAL ATTENDANCE DURING CHICK REARING ----

##~~~~~~~~~~~~~~~~~~~~~~~~~~~~~~~~~~~~~~~~~~~~~~~~~~~~~~~~~~~~~~~~~~~~~~~~~~~~~~

#FIGURE 5 PROPORTION OF VISITS PARENTS PRESENT AND CHICK AGE AT ST HELENA AND ASCENSION

#ASCENSION DATA TAKEN FROM STONEHOUSE (1962)

ggplot(dframe4) +

geom_segment( aes(x=AGEGROUP, xend=AGEGROUP, y=PERCPRESENTSTH, yend=PERCPRESENTASC), color="grey", size=1) +

geom_point( aes(x=AGEGROUP, y=PERCPRESENTSTH, color="PERCPRESENTSTH"), size=4, alpha=0.7 ) +

geom_point( aes(x=AGEGROUP, y=PERCPRESENTASC,color="PERCPRESENTASC") , size=4, alpha=0.7 ) +

scale_y_continuous(name="Proportion of visits parents present (%)", breaks =seq(0,100,25),expand=c(0,0), limits=c(-2,100))+

scale_x_continuous(name="Chick age group (days)", breaks =seq(0,12,1),expand=c(0,0),limits=c(0.5,12.5),labels = c("","0-5", "6-10","11-20","21-30","31-40","41-50","51-60","61-70","71-80","81-90","91-100","101-110"))+

scale_color_manual(values = c("orange", "deepskyblue4"), guide = guide_legend(),labels = c("Ascension", "St Helena") ,name = "") +

theme_ipsum(grid=FALSE, axis=TRUE,

axis_title_size = 14,

base_size = 12,

axis_title_face = "bold",

axis_title_just = "mc",

axis_col = "black",

ticks=TRUE)+

theme(legend.position=c(0.85,0.9),legend.text=element_text(size=14, colour="black"))

##~~~~~~~~~~~~~~~~~~~~~~~~~~~~~~~~~~~~~~~~~~~~~~~~~~~~~~~~~~~~~~~~~~~~~~~~~~~~~~

## FIGURE A2 ADULT MASS VS. CHICK AGE ----

##~~~~~~~~~~~~~~~~~~~~~~~~~~~~~~~~~~~~~~~~~~~~~~~~~~~~~~~~~~~~~~~~~~~~~~~~~~~~~~

#FIGURE A2 ADULT BODY CONDITION DURING EACH WEEK OF CHICK DEVELOPMENT

#SAMPLE SIZES AT THE TOP

ggplot(dframe5,aes(x=WEEKOFCRF, y=Weight))+

geom_boxplot( fill="lightgrey")+

stat_summary(fun.y=mean, geom="point", shape=21, size=3, color="black", fill="white") +#add mean of each year as point

scale_y_continuous(breaks=seq(550,900,50), limits = c(550,930), expand=c(0, 0)) +

geom_text(data=CRmass,aes(WEEKOFCR, Inf, label=n), colour="black", vjust=1,hjust=.5, size=3.5)+

ylab("Adult mass (g)") + xlab("Age of chick (weeks)")+

theme_ipsum(grid=FALSE, axis=TRUE,

axis_title_size = 14,

base_size = 12,

axis_title_face = "bold",

axis_title_just = "mc",

axis_col = "black",

ticks=TRUE)+

theme(legend.position="none")
