# Supplementary material for: Between‐year and spatial variation in body condition across the breeding cycle in a pelagic seabird, the Red‐billed Tropicbird
Source: Ecol Evol. 2023 Dec 27;13(12):e10743. doi: 10.1002/ece3.10743 (PMC10752250; doi:10.1002/ece3.10743)
Supplement: Supplementary file 8 — Data S8. [file ECE3-13-e10743-s010.docx]

##~~~~~~~~~~~~~~~~~~~~~~~~~~~~~~~~~~~~~~~~~~~~~~~~~~~~~~~~~~~~~~~~~~~~~~~~~~~~~~

## --

##------------ RED-BILLED TROPICBIRD CHICK GROWTH ANALYSIS SCRIPT---------------

## --

##~~~~~~~~~~~~~~~~~~~~~~~~~~~~~~~~~~~~~~~~~~~~~~~~~~~~~~~~~~~~~~~~~~~~~~~~~~~~~~

#This is the script used for the analysis of chick grwoth data included in the manuscript entitled

#"Among-year and spatial variation in body condition across the breeding cycle

#in a pelagic seabird, the Red-billed Tropicbird" submitted to Ecology and Evolution Journal

#last updated 04/07/2023

#R studieo version 1.2.5019

##~~~~~~~~~~~~~~~~~~~~~~~~~~~~~~~~~~~~~~~~~~~~~~~~~~~~~~~~~~~~~~~~~~~~~~~~~~~~~~

## INSTALL PACKAGES ----

##~~~~~~~~~~~~~~~~~~~~~~~~~~~~~~~~~~~~~~~~~~~~~~~~~~~~~~~~~~~~~~~~~~~~~~~~~~~~~~

library(RODBC)

library(scales)

library(AICcmodavg)

library(MuMIn)

library(maptools)

library(rgdal)

require(maps)

require(mapdata)

require(maptools)

library(ggmap)

library(hrbrthemes)# for plot formatting

library(move)

require(geosphere)

library(FlexParamCurve)

library(readxl)

library(tidyverse)

library(lubridate)

library(gamm4)

select<-dplyr::select

filter<-dplyr::filter

colcv<-"#fc7d0b"

colst<-"deepskyblue4"

windowsFonts(A = windowsFont("Arial Narrow"))

##~~~~~~~~~~~~~~~~~~~~~~~~~~~~~~~~~~~~~~~~~~~~~~~~~~~~~~~~~~~~~~~~~~~~~~~~~~~~~~

## LOAD DATA ----

##~~~~~~~~~~~~~~~~~~~~~~~~~~~~~~~~~~~~~~~~~~~~~~~~~~~~~~~~~~~~~~~~~~~~~~~~~~~~~~

#SET THE WORKING DIRECTORY

#setwd("G://x")

#READ THE CSV FILES

growth <- read.csv("CHICK_GROWTH.csv")

#SUBSET THE DATA TO LOCATION

growthsth<- subset(growth, LOCATION=="STH")

growthcv<- subset(growth, LOCATION =="CV")

##~~~~~~~~~~~~~~~~~~~~~~~~~~~~~~~~~~~~~~~~~~~~~~~~~~~~~~~~~~~~~~~~~~~~~~~~~~~~~~

## CALCULATE MAXIMUM DAILY MASS GAIN OF CHICKS AT ST HELENA ----

##~~~~~~~~~~~~~~~~~~~~~~~~~~~~~~~~~~~~~~~~~~~~~~~~~~~~~~~~~~~~~~~~~~~~~~~~~~~~~~

nests<-unique(growthsth$CAVITY)

growthsth$WEIGHT<- as.numeric(growthsth$WEIGHT)

growthsth$ESTIMATED_AGE<- as.numeric(growthsth$ESTIMATED_AGE)

massgrowthsth<-data.frame()

for (n in nests){

chickn<-growthsth[growthsth$CAVITY==n,]

chickn<-chickn[!is.na(chickn$WEIGHT),]

agerange<-unique(chickn$ESTIMATED_AGE)

for (a in agerange){

targetmass<-chickn$WEIGHT[chickn$ESTIMATED_AGE==a]

if(a>min(agerange)){ ## no difference for first measurement

originmass<-chickn$WEIGHT[chickn$ESTIMATED_AGE==ap]

diffm<-targetmass-originmass

dailygrwth<-diffm/(a-ap)

out<-data.frame(nest=n,ESTIMATED_AGE=ap,mass1=originmass, mass2=targetmass, increase=diffm, growth=dailygrwth)

massgrowthsth<-rbind(massgrowthsth,out)

} #

ap<-a

} #

} #

massgrowthsth$perc.grwth<-massgrowthsth$growth/massgrowthsth$mass1

massgrowthsth<-massgrowthsth[order(massgrowthsth$perc.grwth,decreasing=T),]

head(massgrowthsth)

tail(massgrowthsth)

summary(massgrowthsth)

#write.table(massgrowth, "st_helena_rbtbchickmassgrowth.csv",row.names=F, sep=',')

##~~~~~~~~~~~~~~~~~~~~~~~~~~~~~~~~~~~~~~~~~~~~~~~~~~~~~~~~~~~~~~~~~~~~~~~~~~~~~~

## CALCULATE MAXIMUM DAILY MASS GAIN OF CHICKS AT CABO VERDE ----

##~~~~~~~~~~~~~~~~~~~~~~~~~~~~~~~~~~~~~~~~~~~~~~~~~~~~~~~~~~~~~~~~~~~~~~~~~~~~~~

nestscv<-as.factor(growthcv$CAVITY)

nestscv<-unique(growthcv$CAVITY)

growthcv$WEIGHT<- as.numeric(growthcv$WEIGHT)

growthcv$ESTIMATED_AGE<- as.numeric(growthcv$ESTIMATED_AGE)

massgrowthcv<-data.frame()

for (n in nestscv){

chickn<-growthcv[growthcv$CAVITY==n,]

chickn<-chickn[!is.na(chickn$WEIGHT),]

agerange<-unique(chickn$ESTIMATED_AGE)

for (a in agerange){

targetmass<-chickn$WEIGHT[chickn$ESTIMATED_AGE==a]

if(a>min(agerange)){ ## no difference for first measurement

originmass<-chickn$WEIGHT[chickn$ESTIMATED_AGE==ap]

diffm<-targetmass-originmass

dailygrwth<-diffm/(a-ap)

out<-data.frame(nest=n,ESTIMATED_AGE=ap,mass1=originmass, mass2=targetmass, increase=diffm, growth=dailygrwth)

massgrowthcv<-rbind(massgrowthcv,out)

}

ap<-a

}

}

massgrowthcv$perc.grwth<-massgrowthcv$growth/massgrowthcv$mass1

massgrowthcv<-massgrowthcv[order(massgrowthcv$perc.grwth,decreasing=T),]

head(massgrowthcv)

tail(massgrowthcv)

summary(massgrowthcv)

#write.table(massgrowth, "cabo_verde_rbtbchickmassgrowth.csv",row.names=F, sep=',')

##~~~~~~~~~~~~~~~~~~~~~~~~~~~~~~~~~~~~~~~~~~~~~~~~~~~~~~~~~~~~~~~~~~~~~~~~~~~~~~

## TEST FOR DIFFERENCES BETWEEN MASS GAIN BETWEEN COLONIES ----

##~~~~~~~~~~~~~~~~~~~~~~~~~~~~~~~~~~~~~~~~~~~~~~~~~~~~~~~~~~~~~~~~~~~~~~~~~~~~~~

#format the data

massgrowthcv<-massgrowthcv %>% mutate(nest=as.numeric(nest)) %>% mutate(colony=ifelse(nest >1, "CABOVERDE","")) %>% mutate(nest=as.character(nest))

massgrowthsth<-massgrowthsth %>% mutate(nest=as.numeric(nest)) %>% mutate(colony=ifelse(nest >0, "STH","")) %>% mutate(nest=as.character(nest))

#combine the two files

masterpercgrowth<-rbind(massgrowthcv,massgrowthsth)

masterpercgrowth$colony<-as.factor(masterpercgrowth$colony)

#model the data using gamm4

model1<-gamm4(perc.grwth~s(ESTIMATED_AGE, by=colony, k=-3)+colony,random = ~(1|nest), family=gaussian(link="identity"), data=masterpercgrowth)

summary(model1$gam)

anova(model1$gam)

plot(model1$gam)

##~~~~~~~~~~~~~~~~~~~~~~~~~~~~~~~~~~~~~~~~~~~~~~~~~~~~~~~~~~~~~~~~~~~~~~~~~~~~~~

## MAKE PREDICTIONS FROM THE GAMM GROWTH MODEL ----

##~~~~~~~~~~~~~~~~~~~~~~~~~~~~~~~~~~~~~~~~~~~~~~~~~~~~~~~~~~~~~~~~~~~~~~~~~~~~~~

# MAKE PREDICTIONS FOR ST HELENA CHICKS

# Step 1: Making a table of prediction data (pdat)

pdat <- expand.grid(ESTIMATED_AGE = seq(2, 106,1), colony="STH");pdat

# Step 2: Making a file containing the predicted data (pred)

pred <- predict(model1$gam, newdata = pdat, na.action = na.exclude, type= "response", se.fit=T);pred

# Step 3: combine the predictions with the predictors into a final dataframe (predframe)

predframe1 <- data.frame (pdat, preds = pred);predframe1

predframe1$upperse <- (predframe1$preds.fit + predframe1$preds.se.fit)

predframe1$lowerse <- (predframe1$preds.fit - predframe1$preds.se.fit)

#DO THE SAME FOR CABO VERDE CHICKS

# Step 1: Making a table of prediction data (pdat)

pdat <- expand.grid(ESTIMATED_AGE = seq(7, 88,1), colony="CABOVERDE");pdat# set to last day calcs done for growth

# Step 2: Making a file containing the predicted data (pred)

pred <- predict(model1$gam, newdata = pdat, na.action = na.exclude, type= "response", se.fit=T);pred

# Step 3: combine the predictions with the predictors into a final dataframe (predframe)

predframe <- data.frame (pdat, preds = pred);predframe

predframe$upperse <- (predframe$preds.fit + predframe$preds.se.fit)

predframe$lowerse <- (predframe$preds.fit - predframe$preds.se.fit)

##~~~~~~~~~~~~~~~~~~~~~~~~~~~~~~~~~~~~~~~~~~~~~~~~~~~~~~~~~~~~~~~~~~~~~~~~~~~~~~

## FIG 7. PROP BODY MASS FLUCTUATIONS IN CHICKS ----

##~~~~~~~~~~~~~~~~~~~~~~~~~~~~~~~~~~~~~~~~~~~~~~~~~~~~~~~~~~~~~~~~~~~~~~~~~~~~~~

#plot of daily proportion of body mass fluctuations with age in red-billed tropicbird chicks

# blue represents St Helena, orange represents Cabo Verde

ggplot()+

geom_point(data=masterpercgrowth, aes(y=perc.grwth, x=ESTIMATED_AGE,colour=colony),alpha=0.6, size=1.5 )+

geom_ribbon(aes(ymin = lowerse, ymax = upperse,x = ESTIMATED_AGE),data=predframe, alpha = 0.5, fill="#fc7d0b" )+

geom_ribbon(aes(ymin = lowerse, ymax = upperse,x = ESTIMATED_AGE),data=predframe1, alpha = 0.5, fill="deepskyblue4" )+

scale_y_continuous(name="Percentage body mass growth (%)", breaks =seq(-0.30,0.6,0.2),expand=c(0,0), limits=c(-0.3,0.5)) +

scale_x_continuous(breaks=seq(0,110,10),limits=c(0,110), expand=c(0,0), name="Estimated age (days)")+

geom_line(data=predframe1, mapping=aes(y=preds.fit, x=ESTIMATED_AGE),col="deepskyblue4", size=1 )+

geom_line(data=predframe, mapping=aes(y=preds.fit, x=ESTIMATED_AGE),col="#fc7d0b", size=1)+

guides(colour=guide_legend(override.aes =list(size = c(5,5),shape=c(16,16), alpha=c(1,1))))+

scale_color_manual(values = c("#fc7d0b", "deepskyblue4"), guide=guide_legend(),labels = c("Cabo Verde", "St Helena") ,name = "") +

theme_ipsum(grid=FALSE, axis=TRUE,

axis_title_size = 14,

base_size = 12,

axis_title_face = "bold",

axis_title_just = "mc",

axis_col = "black",

ticks=TRUE)+

theme(legend.position=c(0.85,0.9),legend.text=element_text(size=12, family = "A",colour="black"))

##~~~~~~~~~~~~~~~~~~~~~~~~~~~~~~~~~~~~~~~~~~~~~~~~~~~~~~~~~~~~~~~~~~~~~~~~~~~~~~

## FIG 6. GROWTH RATES OF RED-BILLED TROPICBIRD CHICKS DURING 2017 ----

##~~~~~~~~~~~~~~~~~~~~~~~~~~~~~~~~~~~~~~~~~~~~~~~~~~~~~~~~~~~~~~~~~~~~~~~~~~~~~~

#TABLE 5 PARAMETER ESTIMATES ALSO GENERATED AS EACH PLOT IS CREATED

#.............................WEIGHT.............................####

growthcvw<-growthcv%>%select(c(1,2,6,9))

growthcvw$WEIGHT<- as.numeric(growthcvw$WEIGHT)

parameter.estimatescb<-data.frame()

summary(growthcvw)

par(mfrow=c(3,2),mar=c(2,4.5,0,1), oma=c(2,0,0,0))

ylabels<-c('Body mass (g)')

for (m in 1){

## FIT GROWTH CURVE ##

modpar(growthcvw$ESTIMATED_AGE, growthcvw[,m+2],pn.options = "massinits")

if(m==1){M1 <- nls((WEIGHT ~ A/((1+m*exp(-k*(ESTIMATED_AGE-i)))^(1/m))), data=growthcvw, start=list(A=massinits$Asym,m=massinits$M,k=massinits$K,i=massinits$Infl))}

outsum<-as.data.frame(summary(M1)$coefficients)

outsum$variable<-names(growthcvw)[m+2]

outsum$param<-row.names(outsum)

parameter.estimatescb<-rbind(parameter.estimatescb,outsum)

## create cabo verde plot with lines

rbtbcv<-growthcvw[,c(m+2,4)]

rbtbcv<-rbtbcv[!is.na(rbtbcv[,1]),]

plot(rbtbcv[,1]~ESTIMATED_AGE, data=rbtbcv, col= alpha(colcv, 0.6),pch=16, cex=0.6, ylim=c(0,1100), xlim=c(0,110), axes=F, frame=F, family = "A", font.lab = 2,xlab="", ylab=ylabels[m], cex.lab=1.4, mgp=c(3.3,0.8,0))

ptrend <- expand.grid(ESTIMATED_AGE = seq(7,90,1));trend <-expand.grid(predict(M1, newdata=ptrend));trends<- data.frame(ptrend,trend)

lines(trends$Var1 ~ trends$ESTIMATED_AGE, lty=1,lwd=2, col="#fc7d0b")

axis(2, at=seq(0,1100,200), labels=T,family = "A", cex.axis=1.2, las=1)

axis(1, at=seq(0,110,10), labels=T,family = "A", cex.axis=1.2)

}

growthw<-growthsth%>%select(c(1,2,6,9))

growthw$WEIGHT<- as.numeric(growthw$WEIGHT)

parameter.estimates<-data.frame()

summary(growthw)

for (m in 1){

## FIT GROWTH CURVE ##

modpar(growthw$ESTIMATED_AGE, growthw[,m+2],pn.options = "massinits")

if(m==1){M1 <- nls((WEIGHT ~ A/((1+m*exp(-k*(ESTIMATED_AGE-i)))^(1/m))), data=growthw, start=list(A=massinits$Asym,m=massinits$M,k=massinits$K,i=massinits$Infl))}

outsum<-as.data.frame(summary(M1)$coefficients)

outsum$variable<-names(growthw)[m+2]

outsum$param<-row.names(outsum)

parameter.estimates<-rbind(parameter.estimates,outsum)

## create st helena plot with lines

rbtbsth<-growthw[,c(m+2,4)]

rbtbsth<-rbtbsth[!is.na(rbtbsth[,1]),]

points(rbtbsth[,1]~ESTIMATED_AGE, data=rbtbsth, col= alpha(colst, 0.6),pch=16, cex=0.6, ylim=c(0,1100), xlim=c(0,110), axes=T, frame=F, xlab="", ylab=ylabels[m], cex.lab=1.4, mgp=c(3.3,0.8,0))#MPG SETS WHERE AXIS LABEL SITS IN RELATION TO GRAPH

ptrend <- expand.grid(ESTIMATED_AGE = seq(2,100,1));trend <-expand.grid(predict(M1, newdata=ptrend));trends<- data.frame(ptrend,trend)

lines(trends$Var1 ~ trends$ESTIMATED_AGE, lty=1,lwd=2, col="deepskyblue4")

}

#..........................WING LENGTH...........................####

growthcvwl<-growthcv%>%select(c(1:3,9))

growthcvwl$WING<- as.numeric(growthcvwl$WING)

parameter.estimatescb<-data.frame()

summary(growthcvwl)

ylabels<-c('Wing length (mm)')

#plot Cavo Verde chick growth

for (m in 1){

## FIT GROWTH CURVE ##

modpar(growthcvwl$ESTIMATED_AGE, growthcvwl[,m+2],pn.options = "massinits")

if(m==1){M1 <- nls((WING ~ A/((1+m*exp(-k*(ESTIMATED_AGE-i)))^(1/m))), data=growthcvwl, start=list(A=massinits$Asym,m=massinits$M,k=massinits$K,i=massinits$Infl))}

outsum<-as.data.frame(summary(M1)$coefficients)

outsum$variable<-names(growthcvwl)[m+2]

outsum$param<-row.names(outsum)

parameter.estimatescb<-rbind(parameter.estimatescb,outsum)

## create plot with lines

rbtbcv<-growthcvwl[,c(m+2,4)]

rbtbcv<-rbtbcv[!is.na(rbtbcv[,1]),]

plot(rbtbcv[,1]~ESTIMATED_AGE, data=rbtbcv,family = "A", font.lab = 2, col= alpha(colcv, 0.6),pch=16, cex=0.6, ylim=c(0,350), xlim=c(0,110), axes=F, frame=F, xlab="", ylab=ylabels[m], cex.lab=1.4, mgp=c(3.3,0.8,0))

ptrend <- expand.grid(ESTIMATED_AGE = seq(7,90,1));trend <-expand.grid(predict(M1, newdata=ptrend));trends<- data.frame(ptrend,trend)

lines(trends$Var1 ~ trends$ESTIMATED_AGE, lty=1,lwd=2, col="#fc7d0b")

axis(2, at=seq(0,350,50), labels=T, family = "A",cex.axis=1.2, las=1)

axis(1, at=seq(0,110,10), labels=T, family = "A",cex.axis=1.2)

}

#plot St Helena chick growth

growthwl<-growthsth%>%select(c(1,2,3,9))

parameter.estimates<-data.frame()

summary(growthwl)

for (m in 1){

## FIT GROWTH CURVE ##

modpar(growthwl$ESTIMATED_AGE, growthwl[,m+2],pn.options = "massinits")

if(m==1){M1 <- nls((WING ~ A/((1+m*exp(-k*(ESTIMATED_AGE-i)))^(1/m))), data=growthwl, start=list(A=massinits$Asym,m=massinits$M,k=massinits$K,i=massinits$Infl))}

outsum<-as.data.frame(summary(M1)$coefficients)

outsum$variable<-names(growthwl)[m+2]

outsum$param<-row.names(outsum)

parameter.estimates<-rbind(parameter.estimates,outsum)

## create plot with lines

rbtbsth<-growthwl[,c(m+2,4)]

rbtbsth<-rbtbsth[!is.na(rbtbsth[,1]),]

points(rbtbsth[,1]~ESTIMATED_AGE, data=rbtbsth,col= alpha(colst, 0.6), pch=16, cex=0.6, ylim=c(0,300), xlim=c(0,110), axes=F, frame=F, xlab="", ylab=ylabels[m], cex.lab=1.4, mgp=c(3.3,0.8,0))

lines(predict(M1, newdat=data.frame(ESTIMATED_AGE=seq(0,100,1))), lty=1,lwd=2, col="deepskyblue4")

}

#..........................CLUMEN LENGTH.........................####

# plot Cabo Verde chick growth

growthcvcl<-growthcv%>%select(c(1,2,7,9))

parameter.estimatescb<-data.frame()

summary(growthcvcl)

ylabels<-c('Culmen length (mm)')

for (m in 1){

## FIT GROWTH CURVE ##

modpar(growthcvcl$ESTIMATED_AGE, growthcvcl[,m+2],pn.options = "massinits")

if(m==1){M1 <- nls((FEATHERS_TO_BILL ~ A/((1+m*exp(-k*(ESTIMATED_AGE-i)))^(1/m))), data=growthcvcl, start=list(A=massinits$Asym,m=massinits$M,k=massinits$K,i=massinits$Infl))}

outsum<-as.data.frame(summary(M1)$coefficients)

outsum$variable<-names(growthcvcl)[m+2]

outsum$param<-row.names(outsum)

parameter.estimatescb<-rbind(parameter.estimatescb,outsum)

## create plot with lines

rbtbcv<-growthcvcl[,c(m+2,4)]

rbtbcv<-rbtbcv[!is.na(rbtbcv[,1]),]

plot(rbtbcv[,1]~ESTIMATED_AGE, data=rbtbcv,family = "A", font.lab = 2,col= alpha(colcv, 0.6), pch=16, cex=0.6, ylim=c(0,60), xlim=c(0,110), axes=F, frame=F, xlab="", ylab=ylabels[m], cex.lab=1.4, mgp=c(3.3,0.8,0))

ptrend <- expand.grid(ESTIMATED_AGE = seq(7,90,1));trend <-expand.grid(predict(M1, newdata=ptrend));trends<- data.frame(ptrend,trend)

lines(trends$Var1 ~ trends$ESTIMATED_AGE, lty=1,lwd=2, col="#fc7d0b")

axis(2, at=seq(0,60,10), labels=T, family = "A",cex.axis=1.2, las=1)

axis(1, at=seq(0,110,10), labels=T,family = "A", cex.axis=1.2)

}

growthcl<-growthsth%>%select(c(1,2,7,9))

parameter.estimates<-data.frame()

summary(growth)

for (m in 1){ #selects the columns 1-6 in csv mimus the first three (m+3) below

## FIT GROWTH CURVE ##

modpar(growthcl$ESTIMATED_AGE, growthcl[,m+2],pn.options = "massinits")

if(m==1){M1 <- nls((FEATHERS_TO_BILL ~ A/((1+m*exp(-k*(ESTIMATED_AGE-i)))^(1/m))), data=growthcl, start=list(A=massinits$Asym,m=massinits$M,k=massinits$K,i=massinits$Infl))}

outsum<-as.data.frame(summary(M1)$coefficients)

outsum$variable<-names(growthcl)[m+2]

outsum$param<-row.names(outsum)

parameter.estimates<-rbind(parameter.estimates,outsum)

## create plot with lines

rbtbsth<-growthcl[,c(m+2,4)]

rbtbsth<-rbtbsth[!is.na(rbtbsth[,1]),]

points(rbtbsth[,1]~ESTIMATED_AGE, data=rbtbsth,col= alpha(colst, 0.6), pch=16, cex=0.6, ylim=c(0,60), xlim=c(0,110), axes=F, frame=F, xlab="", ylab=ylabels[m], cex.lab=1.4, mgp=c(3.3,0.8,0))#MPG SETS WHERE AXIS LABEL SITS IN RELATION TO GRAPH

lines(predict(M1, newdat=data.frame(ESTIMATED_AGE=seq(0,100,1))), lty=1,lwd=2, col="deepskyblue4") # SET WHERE TO STOP THE PREDICTION LINE

}

#......................HEAD AND BILL LENGTH......................####

growthcvh<-growthcv%>%select(c(1,2,5,9))

parameter.estimatescv<-data.frame()

summary(growthcvh)

ylabels<-c('Head & bill length (mm)')

for (m in 1){ #selects the columns 1-6 in csv mimus the first three (m+3) below

## FIT GROWTH CURVE ##

modpar(growthcvh$ESTIMATED_AGE, growthcvh[,m+2],pn.options = "massinits")

if(m==1){M1 <- nls((HEAD_AND_BILL ~ A/((1+m*exp(-k*(ESTIMATED_AGE-i)))^(1/m))), data=growthcvh, start=list(A=massinits$Asym,m=massinits$M,k=massinits$K,i=massinits$Infl))}

outsum<-as.data.frame(summary(M1)$coefficients)

outsum$variable<-names(growthcvh)[m+2]

outsum$param<-row.names(outsum)

parameter.estimatescv<-rbind(parameter.estimatescv,outsum)

## create plot with lines

rbtbcv<-growthcvh[,c(m+2,4)]

rbtbcv<-rbtbcv[!is.na(rbtbcv[,1]),]

plot(rbtbcv[,1]~ESTIMATED_AGE, data=rbtbcv, family = "A", font.lab = 2, col= alpha(colcv, 0.6), pch=16, cex=0.6, ylim=c(0,130), xlim=c(0,110), axes=F, frame=F, xlab="", ylab=ylabels[m], cex.lab=1.4, mgp=c(3.3,0.8,0))#MPG SETS WHERE AXIS LABEL SITS IN RELATION TO GRAPH

ptrend <- expand.grid(ESTIMATED_AGE = seq(7,90,1));trend <-expand.grid(predict(M1, newdata=ptrend));trends<- data.frame(ptrend,trend)

lines(trends$Var1 ~ trends$ESTIMATED_AGE, lty=1,lwd=2, col="#fc7d0b")

axis(2, at=seq(0,130,20), labels=T, family = "A", cex.axis=1.2, las=1)

axis(1, at=seq(0,110,10), labels=T, family = "A", cex.axis=1.2)

}

growthh<-growthsth%>%select(c(1,2,5,9))

parameter.estimates<-data.frame()

summary(growthh)

ylabels<-c('Head and bill length (mm)')

for (m in 1){

## FIT GROWTH CURVE ##

modpar(growthh$ESTIMATED_AGE, growthh[,m+2],pn.options = "massinits")

if(m==1){M1 <- nls((HEAD_AND_BILL ~ A/((1+m*exp(-k*(ESTIMATED_AGE-i)))^(1/m))), data=growthh, start=list(A=massinits$Asym,m=massinits$M,k=massinits$K,i=massinits$Infl))}

outsum<-as.data.frame(summary(M1)$coefficients)

outsum$variable<-names(growthh)[m+2]

outsum$param<-row.names(outsum)

parameter.estimates<-rbind(parameter.estimates,outsum)

## create plot with lines

rbtbsth<-growthh[,c(m+2,4)]

rbtbsth<-rbtbsth[!is.na(rbtbsth[,1]),]

points(rbtbsth[,1]~ESTIMATED_AGE, data=rbtbsth,col= alpha(colst, 0.6), pch=16, cex=0.6, ylim=c(0,130), xlim=c(0,110), axes=F, frame=F, xlab="", ylab=ylabels[m], cex.lab=1.4, mgp=c(3.3,0.8,0))#MPG SETS WHERE AXIS LABEL SITS IN RELATION TO GRAPH

lines(predict(M1, newdat=data.frame(ESTIMATED_AGE=seq(0,100,1))), lty=1,lwd=2, col="deepskyblue4") # SET WHERE TO STOP THE PREDICTION LINE

}

#........................NOSTRIL TO BILL.........................####

#PLOT ONLY FOR ST HELENA

growthn<-growthsth%>%select(c(1,2,8,9))

parameter.estimates<-data.frame()

summary(growthn)

ylabels<-c('Nostril to bill (mm)')

for (m in 1){

## FIT GROWTH CURVE ##

modpar(growthn$ESTIMATED_AGE, growthn[,m+2],pn.options = "massinits")

if(m==1){M1 <- nls((NOSTRIL_TO_BILL ~ A/((1+m*exp(-k*(ESTIMATED_AGE-i)))^(1/m))), data=growthn, start=list(A=massinits$Asym,m=massinits$M,k=massinits$K,i=massinits$Infl))}

outsum<-as.data.frame(summary(M1)$coefficients)

outsum$variable<-names(growthn)[m+2]

outsum$param<-row.names(outsum)

parameter.estimates<-rbind(parameter.estimates,outsum)

## create plot with lines

rbtbsth<-growthn[,c(m+2,4)]

rbtbsth<-rbtbsth[!is.na(rbtbsth[,1]),]

plot(rbtbsth[,1]~ESTIMATED_AGE, data=rbtbsth, family = "A", font.lab = 2,col= alpha(colst, 0.6),pch=16, cex=0.6, ylim=c(0,60), xlim=c(0,110), axes=F, frame=F, xlab="", ylab=ylabels[m], cex.lab=1.4, mgp=c(3.3,0.8,0))

lines(predict(M1, newdat=data.frame(ESTIMATED_AGE=seq(0,100,1))), lty=1,lwd=2, col="deepskyblue4") # SET WHERE TO STOP THE PREDICTION LINE

axis(2, at=seq(0,60,10), labels=T, family = "A",cex.axis=1.2, las=1)

axis(1, at=seq(0,110,10), labels=T,family = "A", cex.axis=1.2)

}

#.............................TARSUS.............................####

#PLOT ONLY FOR ST HELENA

growtht<-growthsth%>%select(c(1,2,4,9))

parameter.estimates<-data.frame()

summary(growtht)

ylabels<-c('Tarsus (mm)')

for (m in 1){ #selects the columns 1-6 in csv mimus the first three (m+3) below

## FIT GROWTH CURVE ##

modpar(growtht$ESTIMATED_AGE, growtht[,m+2],pn.options = "massinits")

if(m==1){M1 <- nls((TARSUS ~ A/((1+m*exp(-k*(ESTIMATED_AGE-i)))^(1/m))), data=growtht, start=list(A=massinits$Asym,m=massinits$M,k=massinits$K,i=massinits$Infl))}

outsum<-as.data.frame(summary(M1)$coefficients)

outsum$variable<-names(growtht)[m+2]

outsum$param<-row.names(outsum)

parameter.estimates<-rbind(parameter.estimates,outsum)

## create plot with lines

rbtbsth<-growtht[,c(m+2,4)]

rbtbsth<-rbtbsth[!is.na(rbtbsth[,1]),]

plot(rbtbsth[,1]~ESTIMATED_AGE, data=rbtbsth, col= alpha(colst, 0.6),pch=16, family = "A", font.lab = 2, cex=0.6, ylim=c(0,45), xlim=c(0,110), axes=F, frame=F, xlab="", ylab=ylabels[m], cex.lab=1.4, mgp=c(3.3,0.8,0))#MPG SETS WHERE AXIS LABEL SITS IN RELATION TO GRAPH

lines(predict(M1, newdat=data.frame(ESTIMATED_AGE=seq(0,100,1))), lty=1,lwd=2, col="deepskyblue4") # SET WHERE TO STOP THE PREDICTION LINE

axis(2, at=seq(0,45,5), labels=T, family = "A",cex.axis=1.2, las=1)

axis(1, at=seq(0,110,10), labels=T,family = "A", cex.axis=1.2)

}

mtext("Age (days)",1,1,outer=T, family = "A", font = 2,cex=1.0)
