# Supplementary material for: Between‐year and spatial variation in body condition across the breeding cycle in a pelagic seabird, the Red‐billed Tropicbird
Source: Ecol Evol. 2023 Dec 27;13(12):e10743. doi: 10.1002/ece3.10743 (PMC10752250; doi:10.1002/ece3.10743)
Supplement: Supplementary file 10 — Appendix S1 and S2. [file ECE3-13-e10743-s008.docx]

# Appendix 1

**Camera protocol and data processing**

Separate infra-red motion sensor cameras (Bushnell Trophy Cameras) were positioned in front of the entrance of each cavity monitored to enable the recording of adult attendance and nesting outcome. Cameras were used in combination with physical observations during the study for a total of 41 nesting attempts from incubation and 13 nesting attempts from the chick rearing phase only, due to limited camera availability in some years (see Table A1 for details). Cameras were programmed to record two consecutive images upon triggering with a minimum interval of 5 seconds between successive triggers. All camera data was processed manually or using Bushnell Scouting Assistant (1.6.0.3, 2017). Whilst viewing the camera pictures, we noted the date and time (with 1 minute accuracy) when focal individuals identified by their unique colour mark combination on their head feathers (under licence by the British Trust for Ornithology (BTO S5526)) entered/exited the nesting cavity. Observations of eggs, chicks and possible predators were also noted to aid identification of breeding outcome: hatching success: true/false, fledging success: true/false.

**Table A1.** Cavity and nest monitoring methods used at St Helena. Some cavities can contain more than one nest at any one time. INC denotes adults at the incubation breeding stage and CR as adults in the chick rearing stage.

|  | Year | | | | |
| --- | --- | --- | --- | --- | --- |
|  | 2013 | 2014 | 2015 | 2016 | 2017 |
| Number of cavities monitored | 24 | 28 | 28 | 28 | 27 |
| Total nests monitored | 29 | 24 | 24 | 16 | 16 |
| Minimum visual inspection | daily | daily | daily | Mon, Wed, Fri | Mon, Wed, Fri |
| Camera deployment details | n/a | 4 INC, 7 CR | 5 INC, 6 CR | All nests (13 cavities),11 frequently used cavities | All frequently used cavities for whole monitoring period. |

# Appendix 2.

**Table A1.** Mean (± s.d) duration of incubation shifts in Red-billed Tropicbirds from 37 monitored nests at St Helena 2013-2017.

| **Shift** | **Sex^1^** | **Duration (days)** | **Range** | **n** |
| --- | --- | --- | --- | --- |
| First | F | 1.40 ± 1.44 | 0.04-6.99 | 27 |
| Second | M | 6.75 ± 1.96 | 2.07-11.0 | 33 |
| Third | F | 6.94 ± 2.10 | 2.00-11.8 | 33 |
| Fourth | M | 7.60 ± 2.28 | 3.01-11.8 | 33 |
| Fifth | F | 7.26 ± 2.33 | 0.95-11.9 | 31 |
| Sixth | M | 6.73 ± 2.24 | 2.57-11.6 | 31 |
| Seventh | F | 5.93 ± 1.64 | 2.85-8.92 | 17 |
| Eighth | M | 4.62 ± 2.72 | 1.61-10.6 | 11 |
| Ninth | F | 3.34 ± 1.98 | 1.13-4.97 | 3 |
| Tenth | M | 2.03 |  | 1 |
| Eleventh | F | 2 |  | 1 |

^1^ F = female, M = male.

**Table A2.** Proportion of Red-billed Tropicbird nest monitoring visits to chicks where at least one parent was present with chick at St Helena between 2013-2017.

| Chick age (weeks) | Total visits | % parents present |
| --- | --- | --- |
| 1 | 265 | 92.08 |
| 2 | 224 | 83.04 |
| 3 | 172 | 68.60 |
| 4 | 134 | 35.07 |
| 5 | 110 | 50.00 |
| 6 | 86 | 33.72 |
| 7 | 76 | 36.84 |
| 8 | 66 | 18.18 |
| 9 | 51 | 23.53 |
| 10 | 39 | 2.56 |
| 11 | 38 | 5.26 |
| 12 | 39 | 2.56 |
| 13 | 35 | 5.71 |
| 14 | 8 | 12.50 |


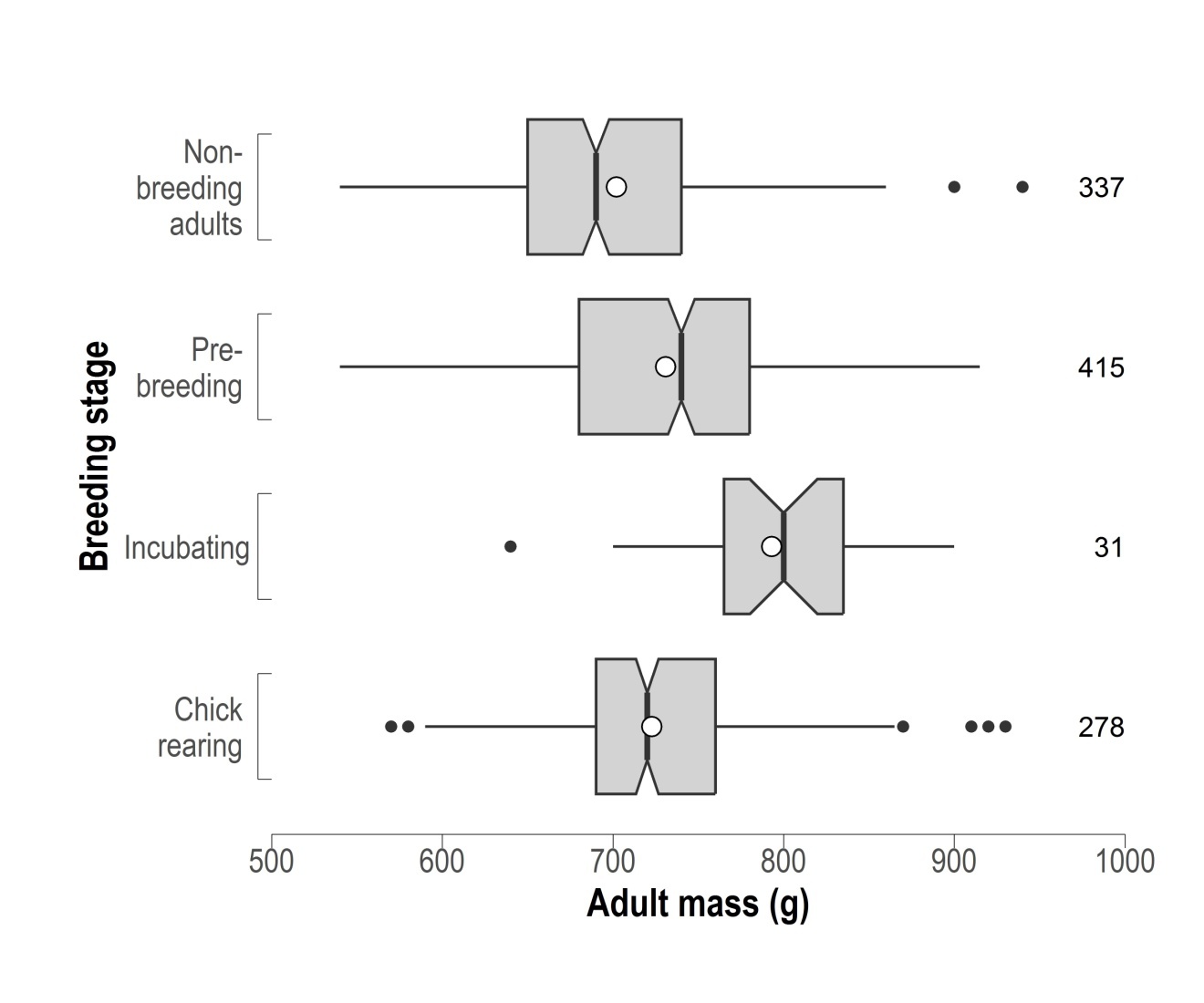


**Figure A1.** Body condition (mass in g) of adult Red-billed Tropicbirds at different breeding stages at St Helena, South Atlantic. Boxplots show median values (thick vertical bar) per breeding stage, including 95% confidence intervals of the median (notches), 25^th^ and 75^th^ percentiles (grey boxes), ranges within 1.5 times the width of the box (horizontal whisker lines), outliers (black points) and means (white points). Sample sizes indicated on the right.


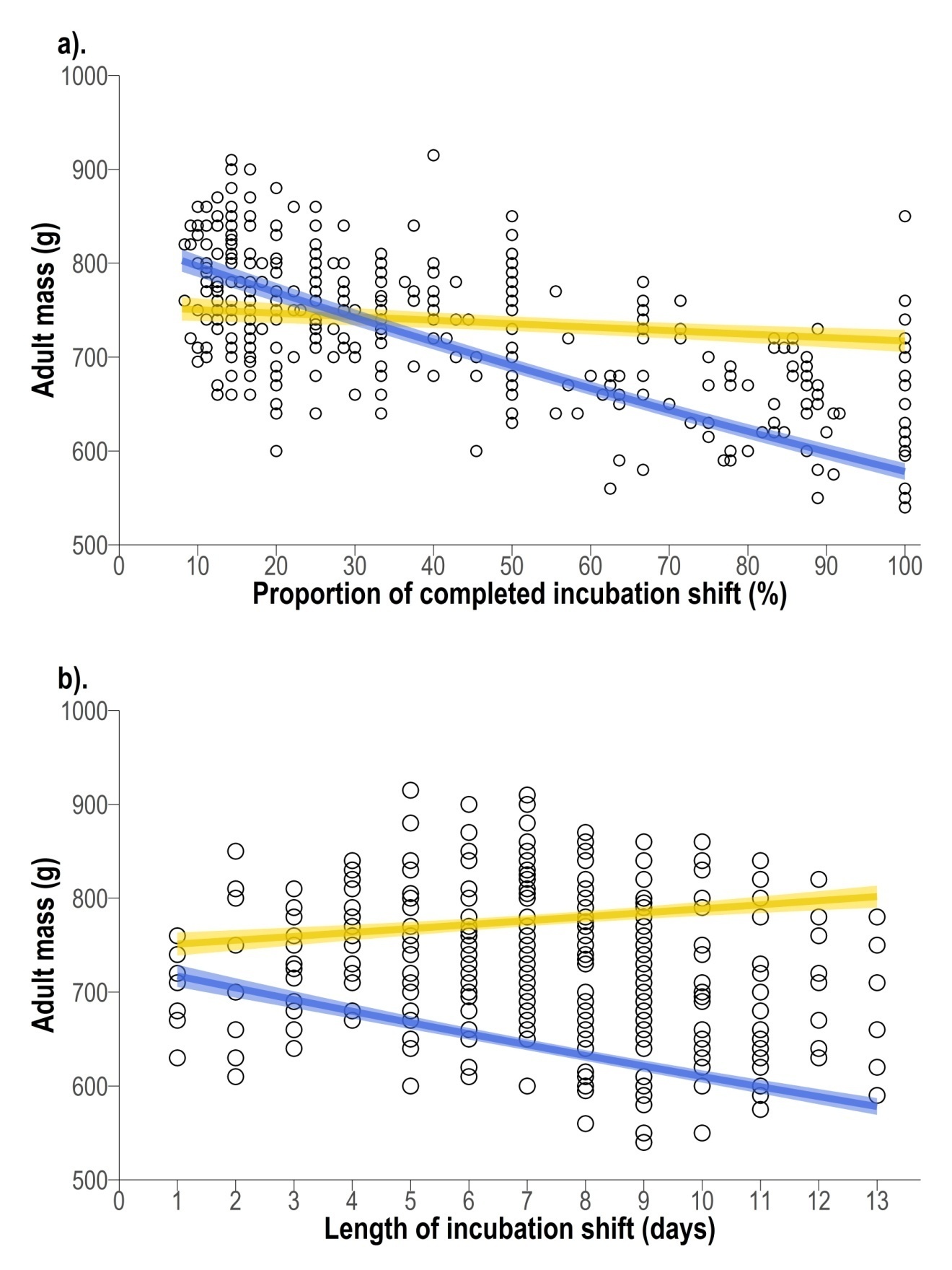


**Figure A2**. Variation in body mass of adult Red-billed Tropicbirds during incubation, showing that body mass is influenced by both the proportion of the incubation shift that has been completed, and the overall length of the incubation shift, and the interaction between these two variables. Open circles represent raw data, solid lines represent model predictions, and shaded areas around the fitted lines represent ± SE. a) Body mass versus proportion of completed incubations shift. The yellow line represents model predictions for the shortest incubation shift (shift duration = 1 day), and the blue line represents the longest incubations shift (13 days). b) Body mass versus incubation shift length (1-13 days). The yellow line represents model predictions for early in the incubation (8.3% of the shift completed), and the blue line represents model predictions for the end of the incubation shift (100% of the incubations shift completed).


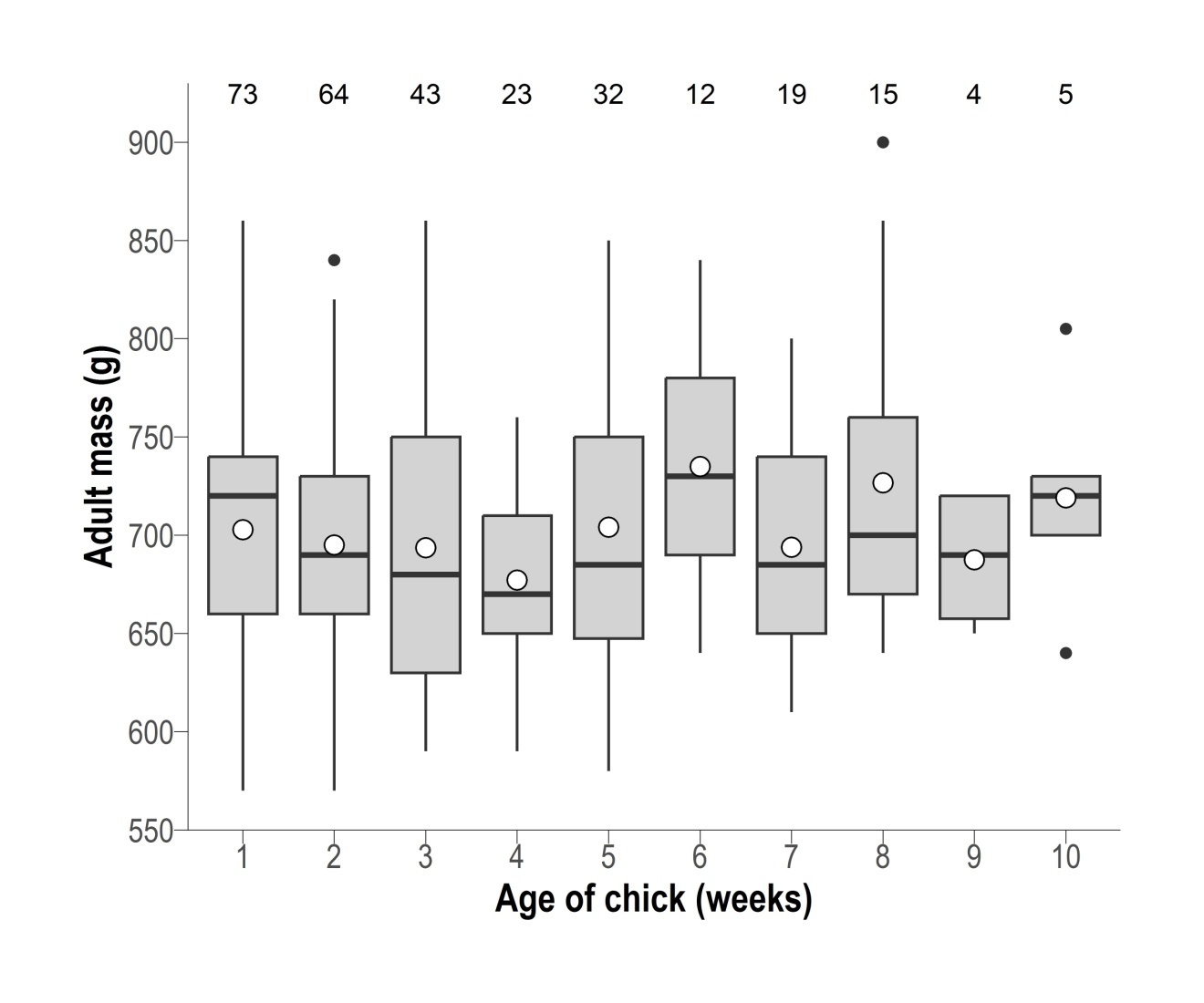


**Figure A3**. Relationship between adult body condition and chick age in weeks. Values above each box are the sample sizes of adults measured in each week of chick development


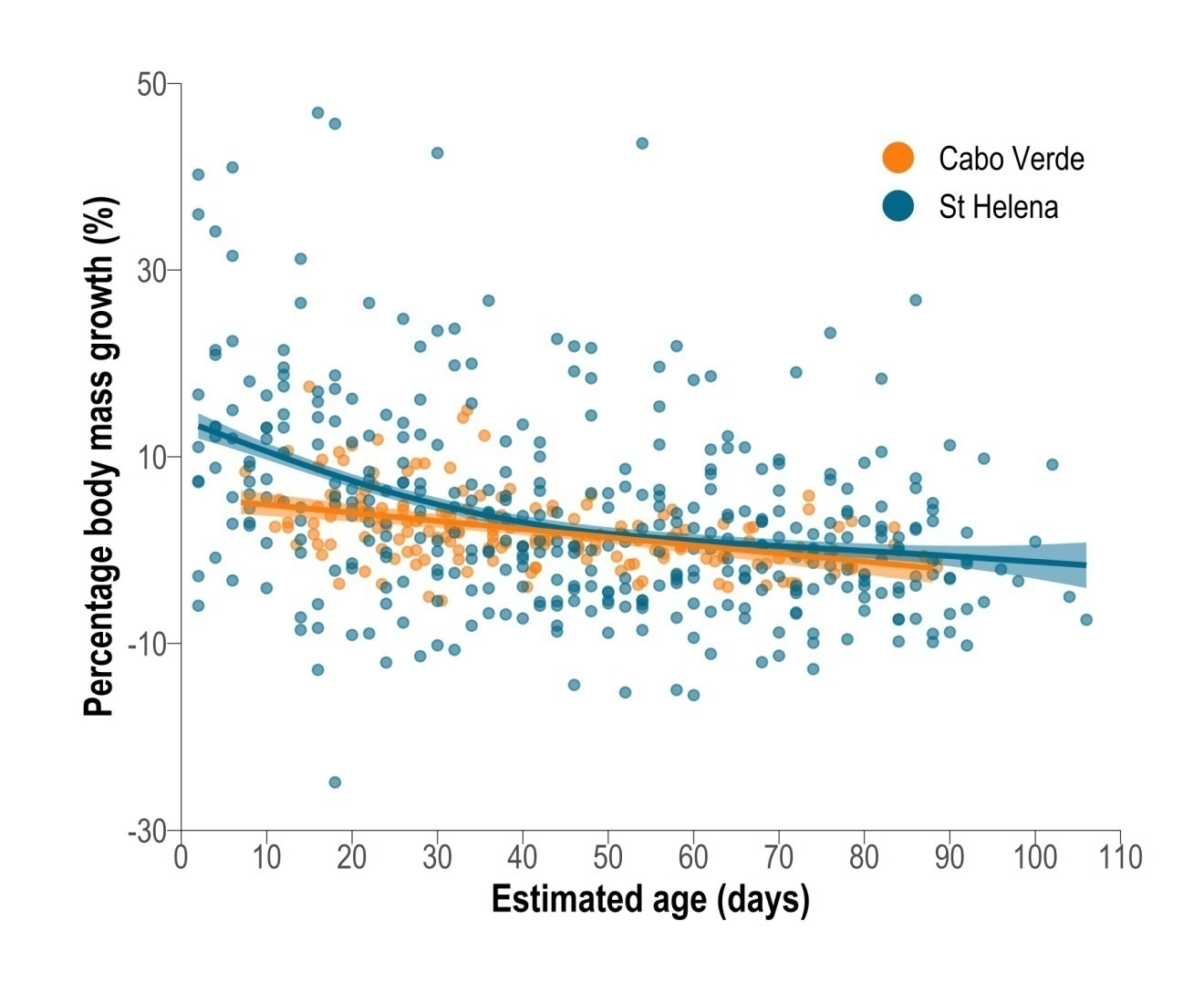


**Figure A4.** Comparison of daily proportion of body mass fluctuations with age in Red-billed Tropicbird chicks from two geographical regions (St Helena: blue lines/circles, Cabo Verde: orange lines/circles) during the 2017 breeding season. Lines are predictions from a GAMM ± 1 SE.
